# *In situ* exsolving RuFe/La_0.6_Sr_0.4_Fe_0.95_Ru_0.05_O_3−δ_ interfaces for direct and ethane-intensified CO_2_ electrolysis in solid oxide electrolysis cells

**DOI:** 10.1093/nsr/nwag265

**Published:** 2026-05-11

**Authors:** Houfu Lv, Yujia Han, Yunfan Fu, Minghao Ma, Yuxiang Shen, Haolin Liu, Hongwu Zhao, Chaobin Zeng, Heng Zheng, Ding Ma, Guoxiong Wang, Xinhe Bao

**Affiliations:** Suzhou National Laboratory, Suzhou 215000, China; State Key Laboratory of Catalysis, iChEM (Collaborative Innovation Center of Chemistry for Energy Materials), Dalian Institute of Chemical Physics, Chinese Academy of Sciences, Dalian 116023, China; Suzhou National Laboratory, Suzhou 215000, China; State Key Laboratory of Catalysis, iChEM (Collaborative Innovation Center of Chemistry for Energy Materials), Dalian Institute of Chemical Physics, Chinese Academy of Sciences, Dalian 116023, China; State Key Laboratory of Catalysis, iChEM (Collaborative Innovation Center of Chemistry for Energy Materials), Dalian Institute of Chemical Physics, Chinese Academy of Sciences, Dalian 116023, China; University of Chinese Academy of Sciences, Beijing 100049, China; State Key Laboratory of Catalysis, iChEM (Collaborative Innovation Center of Chemistry for Energy Materials), Dalian Institute of Chemical Physics, Chinese Academy of Sciences, Dalian 116023, China; University of Chinese Academy of Sciences, Beijing 100049, China; State Key Laboratory of Catalysis, iChEM (Collaborative Innovation Center of Chemistry for Energy Materials), Dalian Institute of Chemical Physics, Chinese Academy of Sciences, Dalian 116023, China; State Key Laboratory of Catalysis, iChEM (Collaborative Innovation Center of Chemistry for Energy Materials), Dalian Institute of Chemical Physics, Chinese Academy of Sciences, Dalian 116023, China; University of Chinese Academy of Sciences, Beijing 100049, China; State Key Laboratory of Catalysis, iChEM (Collaborative Innovation Center of Chemistry for Energy Materials), Dalian Institute of Chemical Physics, Chinese Academy of Sciences, Dalian 116023, China; University of Chinese Academy of Sciences, Beijing 100049, China; Hitachi High-tech (Shanghai) Co., Ltd., Shanghai 200120, China; State Key Laboratory of Porous Materials for Separation and Conversion, Southwest Research & Design Institute of the Chemical Industry, Sichuan 610225, China; Beijing National Laboratory for Molecular Sciences, New Cornerstone Science Laboratory, College of Chemistry and Molecular Engineering, Peking University, Beijing 100871, China; State Key Laboratory of Catalysis, iChEM (Collaborative Innovation Center of Chemistry for Energy Materials), Dalian Institute of Chemical Physics, Chinese Academy of Sciences, Dalian 116023, China; Advanced Institute for Future Energy, Shanghai Key Laboratory of Electrochemical and Thermochemical Conversion for Resources Recycling, State Key Laboratory of Porous Materials for Separation and Conversion, iChEM (Collaborative Innovation Center of Chemistry for Energy Materials), Department of Chemistry, Fudan University, Shanghai 200438, China; State Key Laboratory of Catalysis, iChEM (Collaborative Innovation Center of Chemistry for Energy Materials), Dalian Institute of Chemical Physics, Chinese Academy of Sciences, Dalian 116023, China; Advanced Institute for Future Energy, Shanghai Key Laboratory of Electrochemical and Thermochemical Conversion for Resources Recycling, State Key Laboratory of Porous Materials for Separation and Conversion, iChEM (Collaborative Innovation Center of Chemistry for Energy Materials), Department of Chemistry, Fudan University, Shanghai 200438, China

**Keywords:** solid oxide electrolysis cells, direct CO_2_ electrolysis, ethane-intensified CO_2_ electrolysis, electro-thermal coupling catalysis, confined metal/oxide interface

## Abstract

CO_2_ electrolysis in solid oxide electrolysis cells (SOECs) holds promise for renewable energy storage and carbon recycling. However, current catalysts used in SOECs show decent electrochemical performance but limited CO_2_ conversion. Here, *in situ* exsolution process of the confined RuFe nanoparticles anchored on La_0.6_Sr_0.4_Fe_0.95_Ru_0.05_O_3−δ_ perovskite (RuFe/LSFRu) was revealed, and SOEC using RuFe/LSFRu as cathode shows a current density of 2.75 A cm^−2^ and a CO_2_ conversion of 83.4% for direct CO_2_ electrolysis. Furthermore, the ethane-intensified SOEC employing RuFe/LSFRu cathode achieves ethane and CO_2_ conversion of over 95% (CO_2_/C_2_H_6_ = 4) and syngas production of 0.91 L h^−1^ cm^−2^ by integrating dry ethane reforming process with the reverse water-gas shift and electrolysis reactions. *In situ* electrochemical diffuse reflectance infrared Fourier transform spectroscopy and density functional theory calculations reveal that the decomposition of OH* species to produce H_2_ under the electric ‘driving force’ is crucial to the increase in H_2_ selectivity and CO_2_ conversion. These results highlight the superiority of RuFe/LSFRu as bi-functional catalyst for direct and ethane-intensified CO_2_ electrolysis in SOECs.

## Teaser

This work develops an interface-confined RuFe/LSFRu catalyst which catalyzes direct CO_2_ electrolysis and electro-thermal coupling ethane-intensified CO_2_ reduction with high CO_2_ conversion in solid oxide electrolysis cells.

## INTRODUCTION

Electrocatalytic CO_2_ reduction to produce chemicals and fuels is a potentially important pathway towards a net-zero-emission society [1,2]. High-temperature CO_2_ electro-reduction (600–1000°C) into CO in solid oxide electrolysis cell (SOEC) has emerged as an attractive approach for CO_2_ reduction due to their high electrode reaction kinetics and high Faradaic efficiency, which may have potential for practical application [3,4]. Consequently, the efficiency of SOECs for CO_2_ electrolysis is highly dependent on the electro-catalytic activity of the cathodes [5]. Perovskite-type mixed ionic and electronic conductors (ABO_3_ oxide) are considered as promising substitutes for Ni-based metal-ceramics because of their high anti-carbon ability and excellent redox stability, however, they usually show limited CO_2_ electrolysis activity [6]. Hence, numerous studies are focused on enhancing the CO_2_ electro-catalytic activity and stability of perovskites oxides by modification, including bulk doping with multiple elements to introduce lattice defects [7,8], surface modification of active nanoparticles (NPs) [9–11], surface single atom sites [12–14], and nanocomposite engineering [15]. In recent years, remarkable advances have been achieved in CO_2_ electrolysis via SOECs, including the development of advanced cathode materials and the understanding of the electrolysis mechanism [1,16].

Nonetheless, the performance of current laboratory-grade perovskite catalysts fails to meet

the demands of industrial applications, there remain some major challenges in catalyst design and novel electrochemical process development. Besides, most CO_2_ electrolysis studies to date have been carried out mainly focusing on electrochemical performance, and limited attention has been given for the high CO_2_ conversion efficiency. In addition, carbon deposition during direct CO_2_ electrolysis or CO_2_-H_2_O co-electrolysis in SOEC may occur, especially at high current density and high CO_2_ conversion [17]. Therefore, the feasibility of employing SOECs with efficient perovskite catalysts to achieve the complete electro-reduction of CO_2_ has not been explored, motivating a detailed investigation via direct CO_2_ electrolysis or multi-field coupling CO_2_ reduction with high reactant conversion in SOECs to meet the demands of industrial applications.

In this study, La_0.6_Sr_0.4_FeO_3−δ_ (LSF) perovskite oxide was selected as the parent material with high concentration of oxygen vacancies and oxygen exchange capability. The catalyst is customized via *in situ* exsolution from trace Ru doped LSFRu (La_0.6_Sr_0.4_Fe_0.95_Ru_0.05_O_3−δ_), and the *in situ* exsolution process of RuFe alloy NPs and the confined RuFe/LSFRu interfaces were revealed in detail by *in situ* electron microscopy technology. We show that the bi-functional RuFe/LSFRu catalyst exhibited excellent catalytic performance for direct and ethane-intensified CO_2_ electrolysis in SOECs. The SOEC with RuFe/LSFRu cathode achieves a current density of 2.75 A cm^−2^ and a CO_2_ conversion of 83.4% with CO selectivity of near 100% for direct CO_2_ electrolysis at 800°C. Using a simulated CO_2_-rich gas mixture (CO_2_/C_2_H_6_ = 4) in the RuFe/LSFRu cathode of SOEC, an ethane and CO_2_ conversion of over 95% was achieved with CO and H_2_ selectivity of over 93% via the electro-thermal coupling catalytic process, resulting in the high-selectivity production of syngas at a rate of 0.98 L h^−1^ cm^−2^ at an applied current density of 700 mA cm^−2^. *In situ* electrochemical diffuse reflectance infrared Fourier transform spectroscopy (DRIFTS) and density functional theory (DFT) calculations highlight the importance of the interfacial active center to facilitate the CO_2_ and ethane dissociation, and reveal that the decomposition of OH* species to produce H_2_ under the electric “driving force” is energetically more favorable than the formation of H_2_O, leading to an increase in H_2_ selectivity and CO_2_ conversion. This study provides crucial insights into the development of perovskite-based heterogeneous catalysts for efficient CO_2_ conversion in SOECs.

## RESULTS AND DISCUSSION

### Structure and morphological characterizations

**Figure 1.**
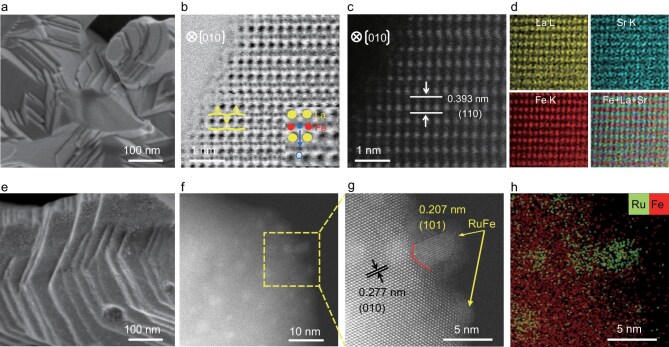
Structural characterizations of LSFRu and RuFe/LSFRu catalysts. (a) SEM image, (b) ABF-STEM image, (c) ADF-STEM image, and (d) STEM-EDS elemental maps of LSFRu. (e) SEM image, (f and g) ADF-STEM images, and (h) STEM-EDS elemental map of RuFe/LSFRu.

XRD and SEM analysis were conducted on the LSFRu and LSF samples before and after reduction (Fig. 1 and Fig. S1). It was observed that the as-prepared LSF perovskite particles presented a spherical morphology with a smooth surface (Fig. S1a). However, SEM image reveals that LSFRu perovskite shows much rougher surface with distinctive and terrace-like microscale structure (Fig. 1a), most probably because of the doping of trace Ru cations. High-resolution scanning transmission electron microscopy (STEM) was employed to provide detailed information of the unique inherent structure of LSFRu. The atomic-scale annular bright field (ABF)-STEM image of LSFRu was viewed along the [010] zone-axis to visualize the O sub-lattice along the Fe-O or Ru-O plane. The ABF image unveils an orderly and regulated crystal structure, and unveils that the inner unit cells present substantial distortions derived from the Jahn-Teller distortion [18] (yellow broken line in Fig. 1b). As shown in the annular dark field (ADF)-STEM image and STEM-energy dispersive spectroscopy (EDS), the La atomic columns share the atomic columns with Sr cations that occupy the A-site, and Fe atomic columns occupy the majority of B-site in perovskite (Fig. 1c and d). Meanwhile, Ru elements were found to be uniformly distributed in the perovskite lattice (Fig. S2). Therefore, we speculate that the doped Ru cations introduce intrinsic defects and thus construct a more terrace-like structure on the surface of perovskite, which is expected to provide abundant nucleation sites for active metal exsolution [19,20].

The result of H_2_ temperature-programmed reduction indicates that LSFRu exhibits two reduction peaks at ∼460 and above 700°C (Fig. S1b). Reduction for a long period of time and a high temperature could result in partial phase transformation due to the excessive metal exsolution, which is consistent with XRD results (Fig. S1c–e). Therefore, reduction at 700°C for 2 h in 5% H_2_/Ar was selected to exsolve structurally stable RuFe/LSFRu interfaces. Figure 1e shows the surface morphology of reduced LSFRu, and high-density metal NPs were exsolved on the terrace fields with the formation of abundant active metal/oxide interfaces. The near invisibility of metallic phase diffraction peak in XRD patterns suggests that small metal NPs are uniformly dispersed on LSFRu surface (Fig. S1c and d). The enlarged STEM image of reduced LSFRu demonstrates that the exsolved metal NPs with an average size of ∼3.5 nm are uniformly distributed in the sample (Fig. 1f). In Fig. 1g, h, and Fig. S3, the ADF-STEM image shows that the exsolved NP with the facet of (101) is anchored on the LSFRu perovskite with the facet of (010). STEM results give clear structural and elemental information at the metal/oxide interface, which indicates that the exsolved RuFe NPs are partially embedded in the substrate to form stable and active hetero interfaces. Meanwhile, the exsolution process is reversible and the surface morphology remains unchanged by further oxidation of RuFe NPs back to the perovskite substrate (Fig. S1f), indicating the redox reversibility of the exsolved RuFe NPs [21,22].

### *In situ* monitoring of NPs exsolution

**Figure 2.**
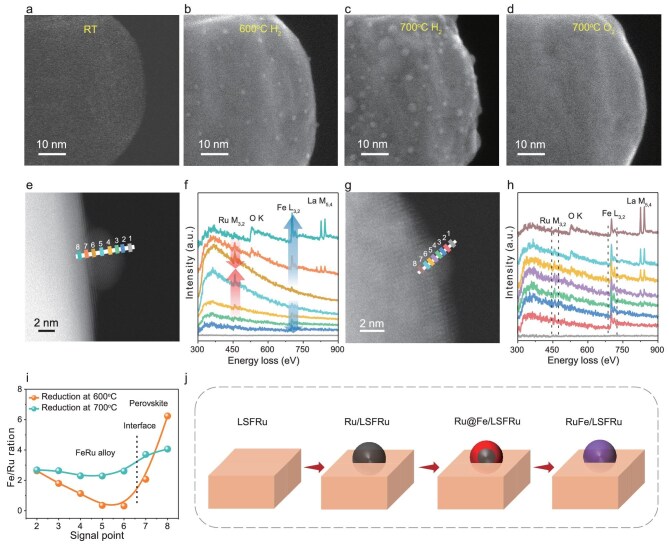
*In situ* monitoring particle exsolution and anchorage during reduction. (a–d) *In situ* SE-STEM images of LSFRu perovskite before reduction, after reduction, and re-oxidation. (e and f) *In situ* ADF-STEM image and STEM-EELS line scan spectra of LSFRu after reduction in H_2_ at 600°C for ∼40 min. (g and h) *In situ* ADF-STEM image and STEM-EELS line scan spectra of LSFRu after reduction in H_2_ at 700°C for ∼30 min. (i) The normalized peak intensity of Fe/Ru bands in (f) and (h). (j) The schematic diagram of the exsolution process of RuFe/LSFRu interface.

*In situ* STEM instrument equipped with a secondary electron (SE) detector has been employed to investigate the *in situ* exsolution of RuFe NPs. Figure 2a–d shows the overall surface morphology of the exsolved NPs via reduction and oxidation treatments. RuFe NPs reversibly move into and out of the perovskite lattice, which may suppress the growth of the metallic NPs and maintain the retention of catalytic activity. Additionally, *in situ* STEM is concomitant with electron energy loss spectroscopy (EELS) to demonstrate the structural and elemental evolution of the newly formed RuFe/LSFRu active sites (Fig. 2e–h). It was observed that Ru cations were preferentially exsolved to form Ru NPs on the perovskite surface due to its low segregation energy [20] (Fig. S4). STEM-EELS spectra show significant Fe L-edge signals in the encapsulation layer on the exsolved Ru NPs during further reduction (Fig. 2e and f). The core-shell encapsulation structure of Ru@Fe may be induced by the strong interactions between metallic Ru and LSF support, which was confirmed via the normalized peak intensity ratio of Fe L-edge to Ru M-edge (Fig. 2i). Uniform RuFe NPs could be assembled via further reduction, and ADF-STEM image and STEM-EELS line scan results further indicate that the exsolved RuFe NPs are deeply embedded in the substrate (Fig. 2g–i). We therefore provide a clear understanding of the *in situ* growth and self-assembly process of the active and stable RuFe/LSFRu interface architectures (Fig. 2j), while the RuFe NPs are regenerated via redox treatments [22].

### CO_2_ electrolysis activity evaluation

An electrolyte-supported SOECs with La_0.8_Sr_0.2_Ga_0.8_Mg_0.2_O_3−δ_ (LSGM) electrolyte, La_0.6_Sr_0.4_Co_0.2_Fe_0.8_O_3−δ_ anode, and LSFRu cathode were assembled to evaluate the CO_2_ electrolysis performance (Fig. S5). Figure S6a shows the linear sweep voltammetry (LSV) curves at 800°C using pure CO_2_ fed to the cathode and air fed to the anode. Apparently, LSFRu cathode exhibits superior CO_2_ electrolysis performance (1.36 A cm^−2^) compared to LSF cathode (1.08 A cm^−2^), indicative of a higher catalytic activity through a simple Ru doping accompanied by the surface terrace-like microscale structure. Furthermore, RuFe/LSFRu cathode exhibits a higher CO_2_ electrolysis performance (1.58 A cm^−2^), and the performance enhancement could be attributed to the *in situ* constructed RuFe/LSFRu active interfaces. Furthermore, CO_2_ electrolysis performance is retained upon switching between oxidizing and reducing conditions, showing the reversible nature of the exsolved RuFe NPs (Fig. S6b). The coarsening of NPs under high temperature reduction leads to a decrease in interfacial sites (Fig. S6c), and excessive reduction results in the destruction of the perovskite structure (Fig. S1e), thus leading to low CO_2_ electrolysis performance (Fig. S6a).

**Figure 3.**
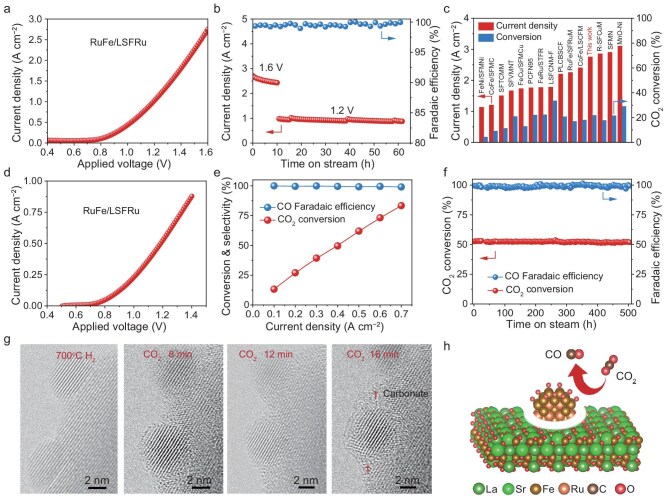
Direct CO_2_ electrolysis evaluation of SOEC with RuFe/LSFRu cathode. (a) LSV curve for direct CO_2_ electrolysis. (b) CO_2_ electrolysis stability test at 1.2 and 1.6 V. (c) Comparison of current density for CO_2_ electrolysis using different cathode catalysts (Table S1). (d) LSV curve for direct CO_2_ electrolysis under a diluted CO_2_ feed gas (5.0 mL cm^−2^, 60% CO_2_ in Ar). (e) CO_2_ conversion and CO Faradaic efficiency at different current densities. (f) Time-dependent CO_2_ conversion and CO Faradaic efficiency at a constant current density of 400 mA cm^−2^. (g) *In situ* BF-STEM images and STEM-EELS spectra of the RuFe/LSFRu interfaces before and after CO_2_ treatment. (h) Schematic illustration for CO_2_ activation at the RuFeO/LSFRu interface.

The RuFe/LSFRu cathode (reduction at 700°C for 2 h in 5% H_2_/Ar) achieved superior electrochemical performance through a simple electrolyte thinning strategy. An SOEC using LSGM electrolyte with a thickness of ∼200 μm and RuFe/LSFRu cathode achieved an industrial-scale current density of 2.75 A cm^−2^ for CO_2_ electrolysis at cell voltage of 1.6 V at 800°C (Fig. 3a and Fig. S7). In addition, SOEC with RuFe/LSFRu cathode obtained low area-specific resistance values benefitting from optimization of the electrode and electrolyte. For example, the ohmic resistance and polarization resistance values are only 0.188 and 0.03 Ω cm^2^ at 1.6 V (Fig. S8a). Figure S8b shows the short-term stability under various applied voltages, and the RuFe/LSFRu cathode maintains stable operation at a high current density of over 2.0 A cm^−2^. Besides, the corresponding Faradaic efficiency of CO remains close to 100%, and thus CO is the sole product in SOEC.

Stability is an important index to evaluate the feasibility of CO_2_ electrolysis, particularly in high-rate systems with industrial-scale current density [1]. To evaluate the long-term stability of RuFe/LSFRu, we conducted tests under a constant applied voltage of 1.6 and 1.2 V (Fig. 3b). The RuFe/LSFRu catalyst demonstrated a satisfactory 10 h stability at ∼2.5 A cm^−2^ and a high 50 h stability at ∼1 A cm^−2^, accompanied by a CO Faradaic efficiency that was close to 100% throughout the operation. Furthermore, CO production rates and corresponding CO_2_ per-pass conversion were also evaluated for assessment of our catalyst. RuFe/LSFRu cathode exhibited a high CO production rate of 20.7 mL min^−1^ cm^−2^ and a conversion of 21.7% at 1.6 V. SOEC with RuFe/LSFRu cathode can achieve a current density of 2.75 A cm^−2^, ranking among the top performances of many other previously reported electrolyte-supported SOECs for direct CO_2_ electrolysis (Fig. 3c and Table S1). However, per-pass CO_2_ conversion (21.7%) is still insufficient, and it is necessary to realize high CO_2_ conversion to meet the demands of industrial applications.

Therefore, we further evaluated the conversion efficiency of RuFe/LSFRu catalyst to demonstrate its practical applicability. RuFe/LSFRu cathode affords a current density of 0.86 A cm^−2^ under a diluted CO_2_ feed gas (Fig. 3d). The per-pass CO_2_ conversion over RuFe/LSFRu increased to 83.4% at a current density of 700 mA cm^−2^ (Fig. 3e), which is close to the thermodynamic theoretical value [23,24], and the CO Faradaic efficiency remained close to 100%. However, stability test indicates that RuFe/LSFRu-based SOEC becomes deactivated within tens of hours when operating at CO_2_ conversion of 83.4%, which is attributed to carbon deposition on the catalyst as the CO partial pressure is close to the threshold for solid carbon formation (Fig. S9) [23]. Nonetheless, RuFe/LSFRu catalyst demonstrated exceptional long-term stability under a constant CO_2_ conversion of 53.5%, as evidenced by a minor increase in cell voltage from 1.15 to 1.34 V after 520 h of operation (degradation rate was 0.36 mV h^−1^) (Fig. 3f and Fig. S10a). The microstructure of RuFe/LSFRu cathode after stability test for 520 h was also analyzed by SEM (Fig. S10b). The RuFe NPs are still uniformly anchored on the perovskite surface without visible particle agglomeration, and the interfacial embedded structure between RuFe and LSFRu is therefore considered contributing to a high stability for CO_2_ electrolysis. Therefore, the RuFe/LSFRu catalyst exhibited high performance for direct CO_2_ electrolysis in SOEC across key parameters, including industrial-scale current density, considerable CO_2_ per-pass conversion, CO yield and Faradaic efficiency, and operation lifetime.

*In situ* STEM tests were carried out to precisely trace the dynamic CO_2_ adsorption and activation process and locate the inherent active sites under the operating conditions of CO_2_ electrolysis. We fabricated clean RuFe/LSFRu active sites in the STEM chamber, and *in situ* STEM-EELS result confirmed the successful construction of RuFe NPs anchored on LSFRu perovskite via reduction at 700°C. *In situ* CO_2_ adsorption was conducted at 200°C, and amorphous adsorbed species can be observed both on metal surfaces and metal/perovskite interfaces (Fig. 3g and Fig. S11). Further STEM-EELS spectra confirm that the intermediate species CO_*x*_ derived from CO_2_ mainly accumulate at the metal/perovskite interfaces [25], proving that the *in situ* constructed RuFe/LSFRu interfaces are the active sites which are responsible for the high CO_2_ electrolysis performance (Fig. S12). *In situ* STEM and STEM-EELS studies also indicate that CO_2_ adsorption at the RuFe/LSFRu interfaces results in the surface reconstruction and formation of a FeO_*x*_ shell on the RuFe NPs (Fig. 3h).

Based on the *in situ* STEM and the experimental results, the RuFe/LSFRu interface with the encapsulated FeO_x_ shell is speculated to enhance the CO_2_ electrolysis performance and stability [9,11]. This can be further supported by DFT calculations. As shown in Fig. S13, CO_2_ activation at the RuFeO/LSFRu interface is an exothermic process (−0.18 eV) and exhibits a stronger adsorption (−1.95 eV) compared to that at the surface oxygen vacancy of LSFRu (detailed structural information and optimized structures are provided in ‘Computational details’ section and Fig. S13 in the Supporting Information). Furthermore, the CO* desorption energy at the RuFeO/LSFRu interface is significantly lower than that at the perovskite surface, which benefits CO release during CO_2_ electrolysis. The projected density of states further reveals pronounced orbital hybridization between the O 2p and C 2p orbitals of CO_2_ and the Ru 3d and Fe 3d orbitals of RuFeO/LSFRu (Fig. S14), which boosts the CO_2_ activation. These results demonstrate that the interfacial RuFe with FeO_*x*_ shell not only promotes CO_2_ activation and dissociation but also accelerates CO* desorption, resulting in enhanced CO_2_ electrolysis performance in SOECs.

### Electro-thermal coupling catalytic DER activity evaluation

**Figure 4.**
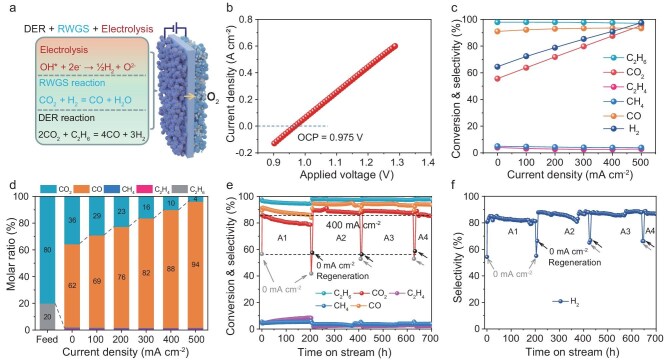
Electro-thermal coupling catalytic DER reaction. (a) Schematic diagram of the electro-thermal coupling catalytic system in SOEC. (b) LSV curve of SOEC with RuFe/LSFRu cathode under a simulated CO_2_-rich feed gas (5.0 mL cm^−2^, 60% CO_2_ and 15% C_2_H_6_ in Ar, gas hourly space velocity = 30 000 mL h^−1^ g^−1^). (c) Conversion of the reactants and selectivity of the products over the RuFe/LSFRu cathode under different current densities. (d) Composition of carbon species in the coupling catalytic system. (e and f) Reactants conversion and products selectivity over the RuFe/LSFRu cathode at 400 mA cm^−2^ for 700 h.

Although a relatively high CO_2_ conversion could be obtained via direct electrolysis in SOEC with the RuFe/LSFRu cathode, carbon is inclined to deposit when the CO partial pressure exceeds the threshold value (∼88%) [23]. To improve the CO_2_ per-pass conversion in SOEC, the strategy of our previously reported tandem electro-thermocatalysis is adopted in the SOEC system [19]. With this approach, the H_2_O formed as a by-product of the reverse water-gas shift (RWGS) reaction would be electrolyzed to form H_2_, helping to shift the thermodynamic equilibrium of the RWGS reaction and achieve high CO_2_ conversion and syngas selectivity [26,27]. We further developed a three-step electro-thermal coupling catalytic ethane reforming process for efficiently converting the simulated CO_2_-rich feed gas (CO_2_/C_2_H_6_ = 4). The key issue in this scheme is the integration of dry ethane reforming (DER) reaction with the RWGS reaction into an oxygen ion-conducting SOEC system, in which water is selectively electrolyzed to shift the equilibrium of the RWGS, promote CO_2_ reduction, and H_2_ and CO production. Dry reforming is a potential process to convert CO_2_ and light alkanes into syngas [28] (H_2_ and CO), which can be subsequently transformed to chemicals and fuels [29]. We used a high CO_2_/ethane ratio (CO_2_/C_2_H_6_ = 4) to achieve high catalytic performance for DER at the RuFe/LSFRu interface (Eq. (1)). Excess CO_2_ would react with H_2_ from the DER to form H_2_O via the RWGS process (Eq. (2)), and the H_2_O formed as a by-product of the RWGS reaction would be electrolyzed to form H_2_, and O^2−^ ions are transported through the electrolyte membrane to the anode to produce O_2_ (Eq. (3)), helping to shift the thermodynamic equilibrium of the RWGS reaction, and achieve high CO_2_ conversion and syngas selectivity (Fig. 4a).


(1) \begin{eqnarray*}
{\mathrm{C}}{{\mathrm{O}}}_{\mathrm{2}} + {{\mathrm{C}}}_{\mathrm{2}}{{\mathrm{H}}}_{\mathrm{6}} = {\mathrm{ 4CO + 3}}{{\mathrm{H}}}_{\mathrm{2}},
\end{eqnarray*}

(2) \begin{eqnarray*}
{{\mathrm{H}}}_{\mathrm{2}}+ {\mathrm{ C}}{{\mathrm{O}}}_{\mathrm{2}}= {\mathrm{ CO }} + {{\mathrm{H}}}_{\mathrm{2}}{\mathrm{O}},
\end{eqnarray*}

(3) \begin{eqnarray*}
{{\mathrm{H}}}_{\mathrm{2}}{\mathrm{O}} = {{\mathrm{H}}}_{\mathrm{2}} + {\mathrm{ 1/2}}{{\mathrm{O}}}_{\mathrm{2}}.
\end{eqnarray*}

Figure 4b shows the LSV curve for the electro-thermal coupling catalytic process over the RuFe/LSFRu cathode. The current density reaches 0.6 A cm^−2^ as the operation voltage increases from open circuit voltage (OCV, 0.975 V) to 1.3 V. At OCP (open circuit potential, thermocatalytic process), the conversion of ethane reached 97.9% and CO_2_ conversion reached 55.6%, which indicates the importance of RuFe/LSFRu for DER and RWGS reactions (Fig. 4c). The H_2_ selectivity in the system was only 64.5%, which is attributed to the RWGS reaction that consumes H_2_ with the formation of H_2_O [30,31]. Ethane conversion remains steady (over 97.0%), and CO_2_ conversion increases rapidly from 55.6% to 95.4% as the current density increased to 0.5 A cm^−2^ (Fig. 4c). Additionally, H_2_ selectivity increased rapidly from 55.6% to 97.5%, indicating that H_2_ was released under the electric ‘driving force.’ In addition, the performance comparison between purely thermal, purely electrochemical, and electro-thermal coupled modes is shown in Fig. S15. These results indicate that ethane reforming and electrolysis reaction need to be operated within a certain high temperature range. The thermo-catalysis process has difficulty achieving complete conversion of CO_2_-rich feed gas, and the electro-thermal coupling catalytic process is a better alternative.

Methane and ethylene by-products that hinder DER were obtained as the minor products (Fig. 4c and d) in the presence of active RuFe NPs [32]. It can be seen from the carbon product distribution result that the content of hydrocarbon products is very low, and the rest are mainly CO and CO_2_ (Fig. 4d). As the current is applied, the final CO content in the system reaches 94% (Fig. 4d), while the H_2_/CO ratio reaches ∼2. This proves that the residual CO_2_ and the formed H_2_O from the RWGS reaction are almost completely electrolyzed with CO and H_2_ released. The decomposition of H_2_O to produce H_2_ at the cathode under the electric ‘driving force’ is the key that leads to an increase in H_2_ selectivity and acceleration of the RWGS process with enhanced CO_2_ conversion.

The catalytic stability of the active RuFe/LSFRu catalyst was investigated for CO and H_2_ production in the electro-thermal coupling catalytic DER system (Fig. 4e and f). The time-on-stream electro-thermal coupling catalytic DER showed relatively stable conversion of ethane and CO_2_ over 200 h at 0.4 A cm^−2^, and slight performance degradation may be attributed to the agglomeration of RuFe NPs. These active NP aggregates can be efficiently regenerated via a simple redox treatment, corresponding to the recovery of catalytic activity [33] (Fig. 4e and f). Meanwhile, redox cycling treatment can enrich the surface Ru elements, thus allowing the exsolution of NPs with higher density upon subsequent reduction [20]. Abundant interfacial active sites ensure the stable operation of electro-thermal coupling catalysis. The ethane and CO_2_ conversion exceeded 97.2% and 85.3%, respectively, while CO and H_2_ selectivity exceeded 93.4% and 84.2%, respectively, when operating for over 700 h. In addition, the applied voltage remained stable throughout the entire process, while the RuFe NPs remained highly dispersed and anchored on the surface of LSFRu perovskite, and the XRD characterization of the post-used catalyst further demonstrates the structural integrity (Fig. S16). Compared to the thermocatalytic DER process, this electro-thermocatalytic coupled DER process has a much higher CO_2_ conversion and H_2_ selectivity. Theoretically, no carbon deposition is expected as the carbon-to-oxygen ratio is less than unity [34]. The carbon balance was over 98.8% and remained nearly unchanged after 700 h, indicating minimal carbon accumulation. Furthermore, the result of Raman spectrum further reveals no carbon accumulation (Fig. S17). The dual functional RuFe/LSFRu catalyst shows great potential for controlled ethane-assisted CO_2_ conversion with high catalytic activity, anti-coking, and redox regeneration properties.

### Electro-thermal coupling catalytic mechanism

**Figure 5.**
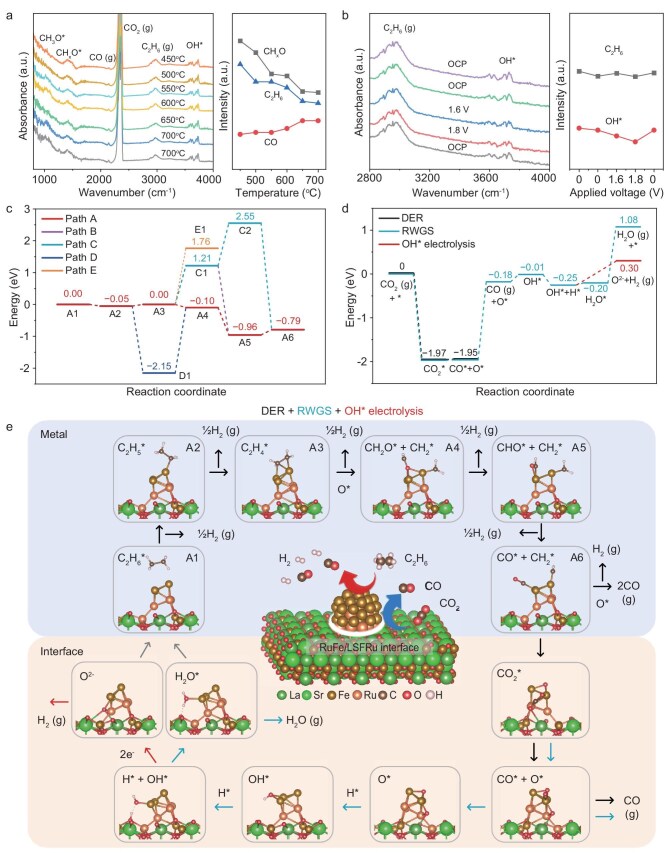
*In situ* DRIFTS spectra and DFT calculations. (a) *In situ* DRIFTS results of RuFe/LSFRu catalyst under 2% C_2_H_6_ + 8% CO_2_ gas. (b) *In situ* electrochemical DRIFTS spectra of RuFe/LSFRu catalyst at 600°C. (c) The energy profiles of C_2_H_6_ activation to produce CO on the RuFe-site of the RuFe/LSFRu catalyst. (d) The reaction paths of DER, RWGS, and OH* electrolysis on the RuFe/LSFRu interface. (e) Schematic illustration for the electro-thermal coupling catalytic DER reaction at the RuFe/LSFRu catalyst. The black, blue and red lines separately represent the process of DER, RWGS and OH* electrolysis.

*In situ* DRIFTS measurements were conducted to identify the key intermediates and monitor the evolution of the dynamic reaction process at the RuFe/LSFRu interfaces (Fig. 5a and Fig. S18). Appreciable bands assigned to gaseous C_2_H_6_ (3000 cm^−1^) and CO_2_ (2360 cm^−1^) could be observed at 400°C. The key CH_3_O* and CH_*x*_O* (or carbonate) were detected at ∼1060 and ∼1460 cm^−1^, respectively [28,35]. When the reaction proceeded from 400 to 700°C, the C_2_H_6_ and CH_*x*_O* (or carbonate) bands decreased progressively, accompanied by the increase in the gaseous CO band. These results show that CO_2_ activation occurs through the redox pathway (CO_2_*→CO*+O*), and the transformation from ethane to CH_*x*_O* with a high proportion of CO_2_ plays a crucial role in ethane activation. The dissociation of CH_*x*_O* and carbonate results in a relatively high intensity of gaseous CO upon increasing the temperature. High CO_2_ concentration promotes the DER and RWGS processes with abundant OH* accumulation. *In situ* electrochemical DRIFTS measurements under the electro-thermal coupling catalytic condition were conducted to monitor the evolution of OH* species with and without the applied voltage (Fig. S18). At 600°C, the intensity of the OH* band decreases upon the applied voltage (Fig. 5b). Once the applied voltage is stopped, the OH* band intensity recovers to the initial one. The *in situ* electrochemical DRIFTS result directly demonstrates OH* consumption under applied voltage, providing the operando mechanistic validation. The OH* species generated via the RWGS reaction serve as a bridge connecting thermocatalytic and electrocatalytic processes, which could be directly electrolyzed during the electro-thermal coupling catalytic process.

DFT calculations were further employed to elucidate the reaction processes on the RuFe/LSFRu interface to gain insight into the catalytic mechanism of the electro-thermal coupling catalytic DER reactions. C_2_H_6_ adsorption and activation were investigated at the RuFe-site of the RuFe/LSFR interface, considering several possible reaction pathways involving oxygen species (O*) derived from CO_2_* dissociation to produce CO* as reported in previous studies [36,37], which are the key steps of DER reaction. As shown in Fig. 5c and Fig. S19, the main reaction pathway is observed at RuFe site via C−H bond cleavage and C=C bond cleavage (C_2_H_6_ (g) → C_2_H_5_* → C_2_H_4_* → CH_2_O* + CH_2_* → CHO* + CH_2_* → CO* + CH_2_*, Path A). The potential-determining step is the process of CHO* → CO* transformation with 0.17 eV energy input, which is significantly smaller than that of the deep dehydrogenation (1.21 and 1.34 eV, Path B and C) and the dehydrogenation process from CH_3_O* to CH_2_O* (2.05 eV, Path D), indicating that C_2_H_6_ preferentially binds with RuFe for C−H bonds dissociation to generate C_2_H_4_* and then oxidized by O* (from CO_2_ activated at interfacial oxygen vacancy) to generate CO, rather than first interacting with O* followed by subsequent dehydrogenation to CH_2_O* and carbon deposition oxidation. In addition, the further cleavage of the C=C bond of C_2_H_4_* was noticeably preferred with an energy release of 0.10 eV, while requiring substantially higher energy input (1.76 eV, Path E, E1) for C_2_H_4_* desorption to C_2_H_4_ (g).

Based on the above thermodynamic analysis, the electro-thermal coupling catalytic DER reactions are illustrated in Fig. 5d and e. C_2_H_6_ undergoes preferential dehydrogenation and oxygenation at the metallic RuFe site, whereas CO_2_ adsorption and dissociation predominantly occur at the RuFe/LSFRu interface. Moreover, the Fe-terminated active sites at the RuFe surface exhibit lower C−H bond dissociation barrier and CO desorption energy than the Ru-terminated surface (Fig. S20). The large amount of hydrogen species (H*) generated from C_2_H_6_ dehydrogenation migrate from the RuFe site to the interface to form hydroxyl intermediates (OH*) through reacting with the oxygen species (O*) derived from excessive CO_2_ dissociation. The OH* intermediates can proceed through two possible pathways: (i) reacting with H* via the RWGS process to form H_2_O (g), or (ii) undergoing electrolysis to yield H_2_ (g) and O^2−^, coupled with a two-electron transfer under an applied voltage. However, in the RWGS process, this step is often regarded as the rate-determining step due to its large energy barrier (typically > 1 eV for most catalysts) [38–40], which is consistent with our calculated energy barrier value of 1.28 eV. In contrast, under the electro-thermal coupling catalytic reaction mode, OH* and H* may preferentially be electrolyzed to yield H_2_ (g) and O^2−^ through a much lower energy barrier (0.55 eV, Fig. 5d), thereby accounting for the enhanced H_2_ generation. Hence, these results demonstrate that the electro-thermal coupled system can effectively promote CO_2_ conversion and syngas selectivity by enabling thermodynamically favorable OH* electrolysis. The electro-thermal conversion simulation results are basically consistent with experimental data. OH* species are electrolyzed to generate H_2_ to promote the RWGS process, which serves as a bridge connecting thermocatalytic and electrocatalytic processes. Therefore, the electrocatalytic and thermocatalytic processes are synergistically coupled rather than a simple series connection of two independent processes.

## CONCLUSION

In this work, we fabricated an active and stable RuFe/LSFRu catalyst via an *in situ* exsolution strategy for direct CO_2_ electrolysis and electro-thermocatalytic DER reaction in SOECs. The RuFe/LSFRu catalyst delivered a single-pass CO_2_ conversion of 83.4% with CO selectivity of nearly 100% for direct CO_2_ electrolysis. Furthermore, we reported the successful development of electro-thermocatalytic ethane assisted CO_2_ reduction over the RuFe/LSFRu cathode, and an ethane and CO_2_ conversion of over 95% were achieved with a CO and H_2_ selectivity of over 93%, resulting in the production of syngas at a rate of 0.98 L h^−1^ cm^−2^ as well as a desirable high stability of 700 h. Based on experimental measurements and theoretical calculations, we present that the metal/oxide interfaces provide multifunctional catalytic sites for thermal and electrocatalytic CO_2_ conversion. OH* species are electrolyzed to generate H_2_ to promote the RWGS process, which serves as a bridge connecting thermocatalytic and electrocatalytic processes. Our work provides valuable insight into the rational design of efficient catalysts and novel electro-thermal coupling catalytic system to develop high-performance SOEC systems for CO_2_ reduction into valuable carbon-based chemicals.

## MATERIALS AND METHODS

Detailed descriptions of materials and methods are presented in the online Supplementary Data.

## Supplementary Material

nwag265_Supplemental_File
