# Supplementary material for: In situ exsolving RuFe/La0.6Sr0.4Fe0.95Ru0.05O3−δ interfaces for direct and ethane-intensified CO2 electrolysis in solid oxide electrolysis cells
Source: Natl Sci Rev. 2026 May 11;13(12):nwag265. doi: 10.1093/nsr/nwag265 (PMC13309935; doi:10.1093/nsr/nwag265)
Supplement: nwag265_Supplemental_File [file nwag265_supplemental_file.pdf]

Supporting Information

In situ exsolving RuFe/$La_{0.6}Sr_{0.4}Fe_{0.95}Ru_{0.05}O_{3-\delta}$ interfaces for direct and ethane-intensified CO_2 electrolysis in solid oxide electrolysis cells

Houfu Lv[1,3,#], Yujia Han[1,3,#], Yunfan Fu[3,4], Minghao Ma[3,4], Yuxiang Shen[3], Haolin Liu[3,4], Hongwu Zhao[3,4], Chaobin Zeng[5], Heng Zheng[6], Ding Ma[7], Guoxiong Wang[2,3,*] and Xinhe Bao[2,3]

[1]Suzhou National Laboratory, Suzhou 215000, China;

[2]Advanced Institute for Future Energy, Shanghai Key Laboratory of Electrochemical and Thermochemical Conversion for Resources Recycling, State Key Laboratory of Porous Materials for Separation and Conversion, iChEM (Collaborative Innovation Center of Chemistry for Energy Materials), Department of Chemistry, Fudan University, Shanghai 200438, China;

[3]State Key Laboratory of Catalysis, iChEM (Collaborative Innovation Center of Chemistry for Energy Materials), Dalian Institute of Chemical Physics, Chinese Academy of Sciences, Dalian 116023, China;

[4]University of Chinese Academy of Sciences, Beijing 100049, China;

[5]Hitachi High-tech (Shanghai) Co., Ltd., Shanghai 200120, China;

[6]State Key Laboratory of Porous Materials for Separation and Conversion, Southwest Research & Design Institute of the Chemical Industry, Sichuan 610225, China;

[7]Beijing National Laboratory for Molecular Sciences, New Cornerstone Science Laboratory, College of Chemistry and Molecular Engineering, Peking University, Beijing 100871, China

***Corresponding authors**. E-mails: wangguoxiong@fudan.edu.cn; wanggx@dicp.ac.cn

[#]Equally contributed to this work.

Experimental and Computational sections

Catalyst synthesis. For the synthesis of 0.03 mol $La_{0.6}Sr_{0.4}Fe_{0.95}Ru_{0.05}O_{3-\delta}$ (LSFRu), 7.5 g of citric acid and 7.5 g of polyvinyl alcohol were added to 300 mL of deionized water, and $La(NO_3)_3{\cdot}6H_2O$, $Sr(NO_3)_2$, $Fe(NO_3)_3{\cdot}9H_2O$, and $RuCl_3$ were then dissolved into the above solution. After evaporation, the LSFRu powder was obtained via calcination at 1000°C for 5 h. $La_{0.6}Sr_{0.4}FeO_{3-\delta}$ (LSF) and $Gd_{0.2}Ce_{0.8}O_{1.9}$ (GDC) powder was synthesized via the same method.

Fabrication of the SOEC system. The LSFRu-GDC|LSGM (FuelCellMaterials)|LSCF-GDC ($La_{0.6}Sr_{0.4}Co_{0.2}Fe_{0.8}O_{3-\delta}$-$Gd_{0.2}Ce_{0.8}O_{1.9}$, SOFCMAN) configuration was used for the CO_2 electrolysis and tandem electro-thermocatalytic dry reforming of ethane (DER) reactions in the solid oxide electrolysis cell (SOEC). LSCF-GDC was used as the classical anode catalyst for the oxygen evolution reaction. LSFRu was used as the multifunctional catalyst after *in situ* exsolution of RuFe alloy nanoparticles (NPs) via H_2 treatment. Effective electrode area: 0.5 cm^2. Catalyst mass: 10 mg.

Performance evaluation. 5.0% N_2 + 15.0% C_2H_6 + 60.0% CO_2 in Ar (5 mL min^{-1}), and 5.0% N_2 + 60.0% CO_2 in Ar were the feed gases for the tandem electro-thermocatalytic DER and direct CO_2 electrolysis reactions in SOECs, and the off gases were analyzed through on-line gas chromatography (Agilent GC490). The electrochemical analyses were performed with a Metrohm Autolab PGSTAT 302 N.

The reactant conversion and product selectivity were calculated as:

$$C_2H_6\ \text{conversion}\ (\%) = \frac{[C_2H_6]_{in} - [C_2H_6]_{out}}{[C_2H_6]_{in}} * 100$$

$$CO_2\ \text{conversion}\ (\%) = \frac{[CO_2]_{in} - [CO_2]_{out}}{[CO_2]_{in}} * 100$$

$$CO\ \text{selectivity}\ (\%) = \frac{[CO]_{out}}{([C_2H_6]_{in} - [C_2H_6]_{out})*2 + [CO_2]_{in} - [CO_2]_{out}} * 100$$

$$H_2\ \text{selectivity}\ (\%) = \frac{[H_2]_{out}}{([C_2H_6]_{in} - [C_2H_6]_{out})*3} * 100$$

Characterizations. X-ray diffraction (XRD) measurements were performed with a PANalytical Empyrean diffractometer. H_2 temperature-programmed reduction (H_2-TPR) measurement was performed using a Micromeritics Chemisorption in 10% H_2/Ar (30 mL min^{-1}) with heating from 30 to 850°C (10°C min^{-1}). *In situ* Scanning Transmission Electron Microscopy (STEM) measurements were performed with a HITACHI HF5000 microscopy (environmental aberration-corrected TEM/STEM/SE) operating at 200 kV with a gas pressure of 10 Pa. The LSFRu catalyst was loaded

onto a micro-electro-mechanical system chip for *in situ* reduction, and the catalytic reactions were run at 200-800°C in 10 Pa H_2 or CO_2 (2 mL min^{-1}). *Ex situ* STEM-EDS (Energy dispersive spectroscopy) elemental maps were collected with a JEM-F200 operated at 200 kV. Scanning electron microscopy (SEM) images were obtained on JSM-7900 system operated at 5 kV. *In situ* diffuse reflectance infrared Fourier transform spectroscopy (DRIFTS) and *in situ* electrochemical DRIFTS experiments were conducted using *in situ* electrochemical reaction cell working at high temperature (Hefei *In Situ* Technology Co. Ltd.) with a Thermo iS50 spectrometer. The testing SOEC was fabricated with a similar configuration of LSFRu-GDC|LSGM|LSCF-GDC. The LSGM electrolyte disk had a diameter of 10 mm, and the sintered electrode area was 5 mm in diameter. The cell was heated to 400 °C in a 5% H_2/Ar flow and reduced for 1 h, after which the feed gas (2% C_2H_6 + 8% CO_2 in N_2, 20 mL min^{-1}) was introduced for the *in situ* DRIFTS measurement at different temperatures. Subsequently, the temperature increased to 600°C and similar *in situ* electrochemical tests were performed using the same feed gas (2% C_2H_6 + 8% CO_2 in N_2, 2 mL min^{-1}).

Computational details. First-principles calculations were carried out using the Vienna *ab initio* Simulation Package (VASP) code that based on density functional theory (DFT)[1]. The generalized gradient approximation (GGA) with the Perdew-Burke-Ernzerhof (PBE) exchange-correlation functional[2-4] was adopted to describe electronic exchange and correlation effects. A plane-wave cutoff energy of 500 eV was used for all calculations. The convergence of the total energy change and the maximum force on each atom were set to 10^{-6} eV and 0.03 eV Å^{-1} during geometry optimization processes. A Monkhorst-Pack *k*-point grid was adopted for Brillouin-zone sampling with a density of about one point per ~0.03 Å^{-3} during the structure optimizations, while a grid of approximately one point per ~0.02 Å^{-3} for electronic property calculations[5]. An effective Hubbard U = 5.5 eV was used for La and 4.0 eV for Fe. A vacuum layer of 15 Å was introduced along the *z*-direction to isolate periodic structure in the slab models. The D3 Grimme dispersion correction was employed to account for van der Waals (vdW) interactions during the tandem electro-thermal coupling catalytic DER progress.

To investigate the CO_2 electrolysis and tandem electro-thermal coupling catalytic DER reaction of LSFRu with oxygen vacancies, FeO_x shell coated on RuFe/LSFRu and RuFe/LSFRu, a series of surface models were constructed based on the XRD and HRTEM results, including LSFRu(010)-V_O (labelled as LSFRu), Ru_3Fe_3O/LSFRu(010)-V_O (labelled as RuFeO/LSFRu) and

Ru_3Fe_3@LSFRu(010)-V_O (labelled as RuFe/LSFRu). LSFRu was constructed based on a p(2×2) cell with two repeat unit layers of $La_{0.5}Sr_{0.5}FeO_3$(010) with one Ru atom and one oxygen vacancy on the surface (4 Ru atoms, 16 La atoms, 16 Sr atoms, 34 Fe atoms, and 95 O atoms). A Fe_3Ru_3 cluster with one oxygen atom was positioned on the surface of LSFRu to simulate the model of the partially oxidized RuFe cluster supported on the perovskite (RuFeO/LSFRu), while that of no oxygen atom was used to simulate the exsolved FeNi cluster supported on the perovskite (RuFe/LSFRu). During the calculations, the bottom unit layer was frozen, while the surface unit layer with the Ru_3Fe_3O or Ru_3Fe_3 cluster and the adsorbate were relaxed.

The adsorption energies (E_{ads}) of CO_2 or H_2O or C_2H_6 on oxygen vacancy or interface or metal were estimated by the following formula:

$$E_{ads} = E_{Gas+support} - E_{support} - E_{Gas}$$

where $E_{Gas+support}$ is the total energy of the support decorated with gas molecule, $E_{support}$ is the total energy of the support, and E_{Gas} is the total energy of gas molecule.

The reaction energies (ΔE) of CO_2 or H_2O or C_2H_6 on oxygen vacancy or interface or metal were estimated by the following formula:

$$\Delta E = E_{FS} - E_{IS}$$

where E_{FS} is the total energy of the support adsorbed with final species, E_{IS} is the total energy of the support adsorbed with initial species.

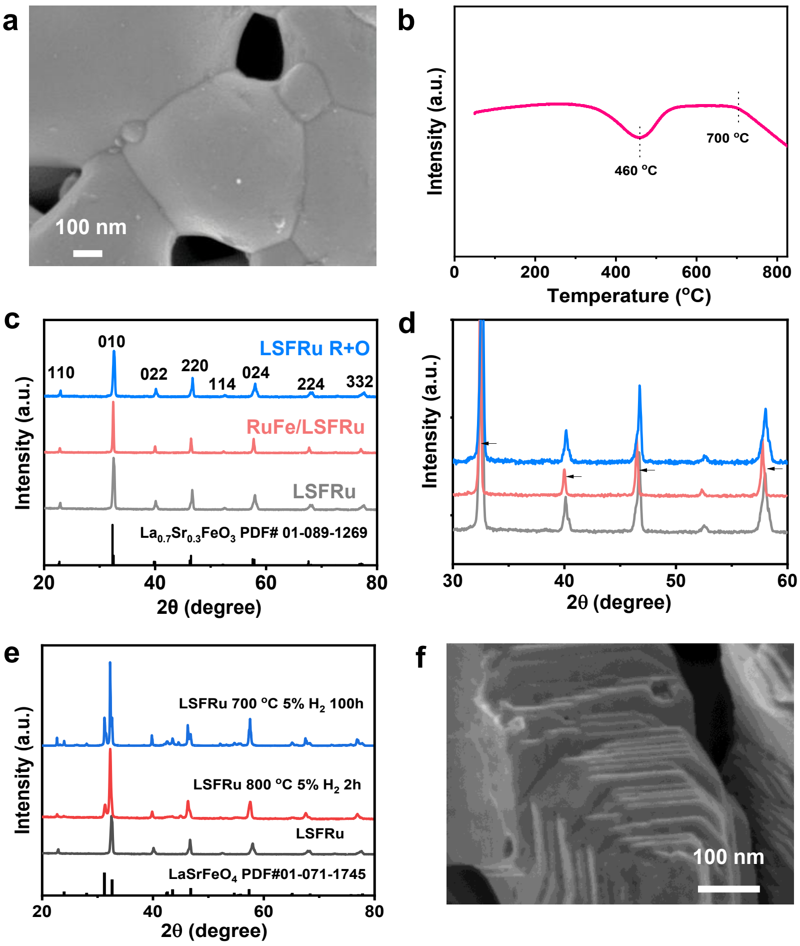

Figure S1. (a) SEM image of the as-prepared LSF sample. (b) H_2-TPR profile of LSFRu. (c-d) XRD patterns of LSFRu, reduced LSFRu (700°C, 5% H_2, 2 h), and re-oxidized LSFRu samples. (e) XRD patterns of LSFRu and reduced LSFRu (800°C, 5% H_2, 2 h and 700°C, 5% H_2, 100 h). (f) SEM image of the re-oxidized LSFRu sample.

The LSFRu sample displays Orthorhombic perovskite structure (Pbnm(62)). Reduction of LSFRu for a longer period of time (700°C, 5% H_2, 100 h) and a higher temperature (800°C, 5% H_2/Ar, 2 h, LSFRu-800) could result in partial phase transformation (Figure S1c-e), reduction at 700°C for 2 h in 5% H_2/Ar was selected to exsolve structurally stable RuFe/LSFRu interfaces.

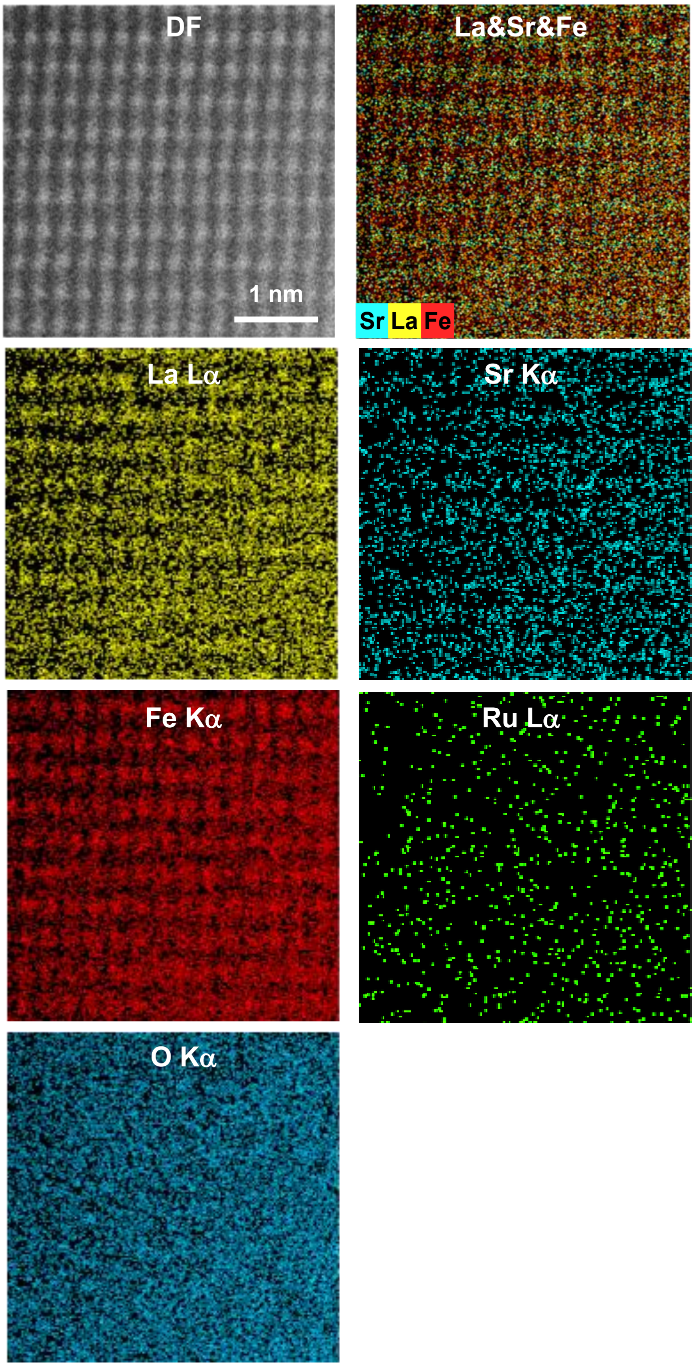

Figure S2. Dark field (DF)-STEM image and STEM-EDS elemental maps of as-prepared LSFRu.

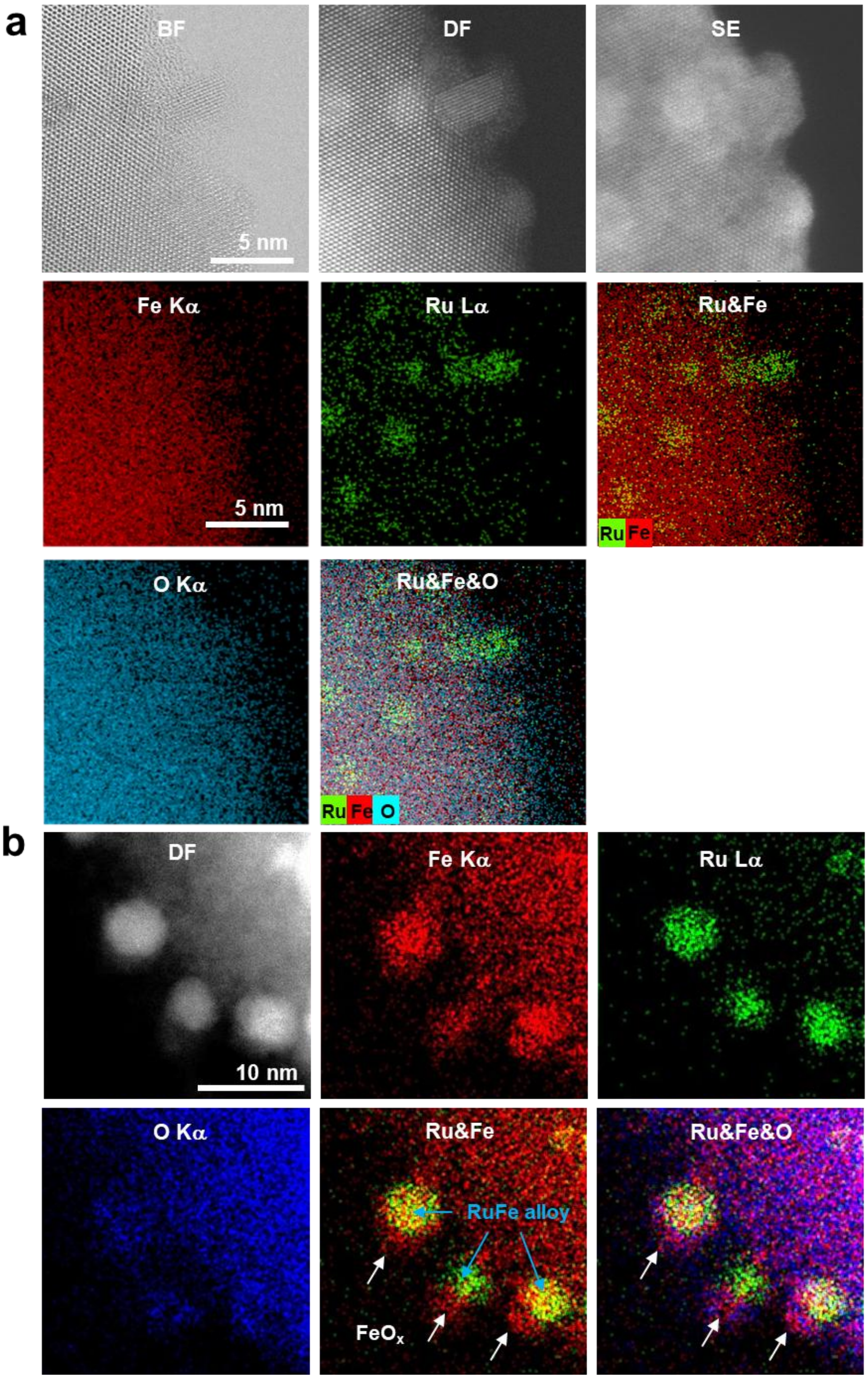

Figure S3. STEM images and STEM-EDS elemental maps of RuFe/LSFRu.

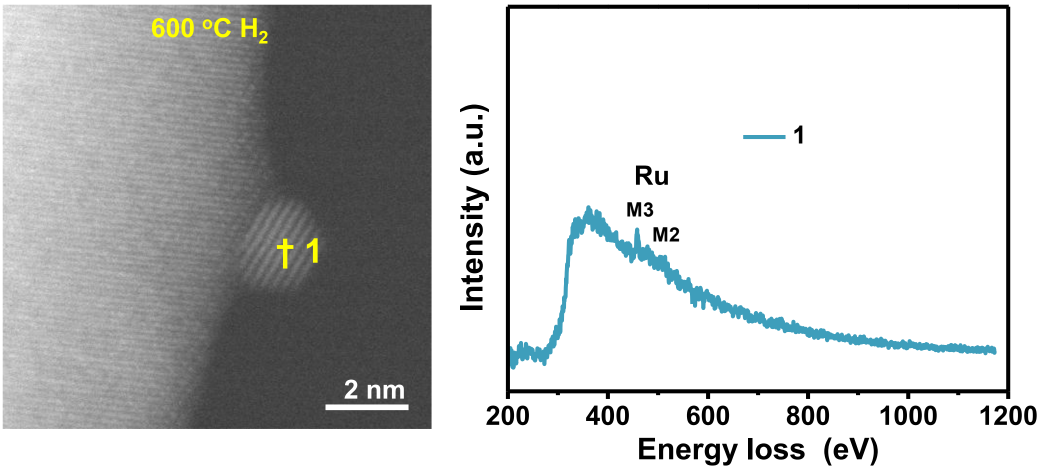

Figure S4. *In situ* DF-STEM image and STEM-EELS spectra of LSFRu after reduction in H_2 at 600°C for ~45 min.

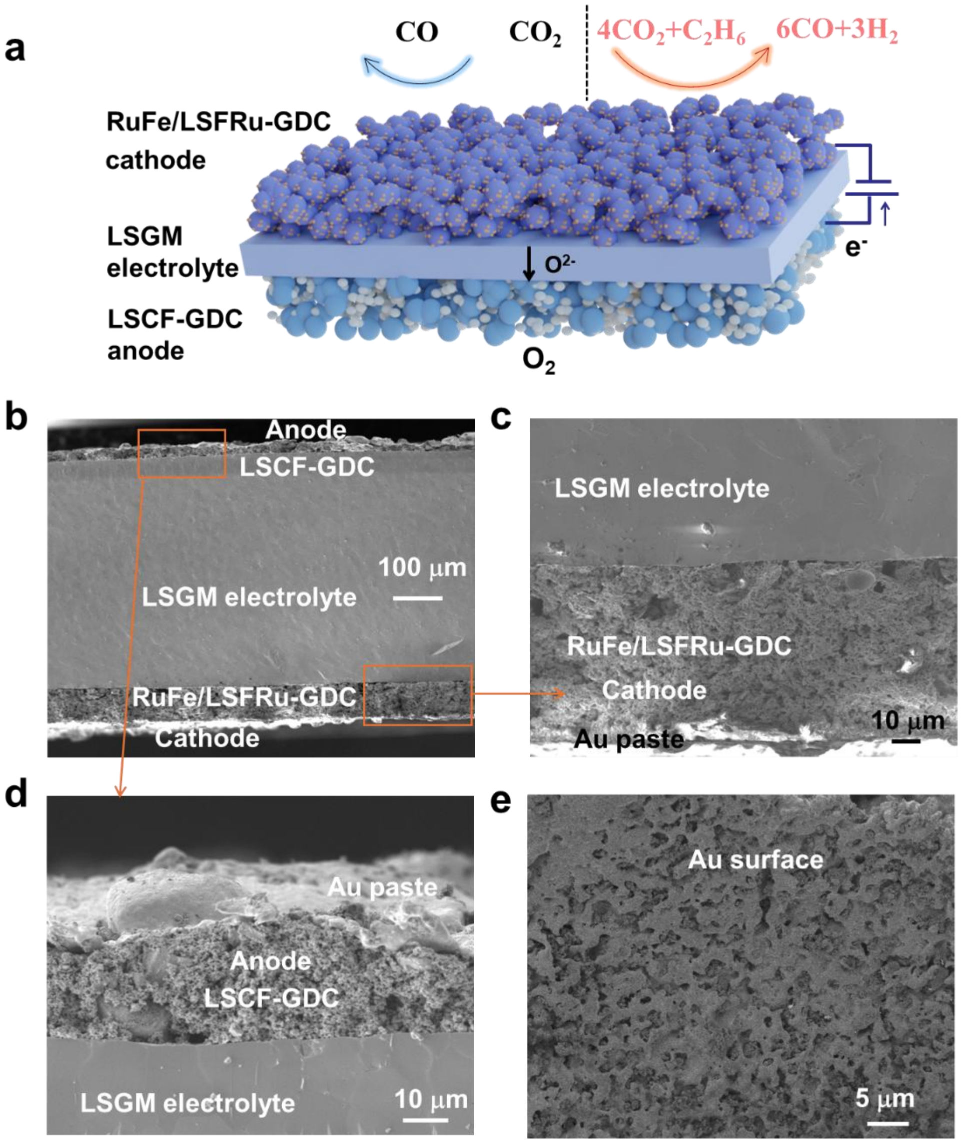

Figure S5. (a) Schematic diagram of the electro-thermal coupling catalytic and direct CO_2 electrolysis system in SOEC. (b) Cross-sectional SEM image of LSFRu-GDC|LSGM|LSCF-GDC cell. (c) Cross-sectional SEM image of LSGM supported LSFRu-GDC cathode. (d) Cross-sectional SEM image of LSGM supported LSCF-GDC anode. (e) SEM image of the surface of LSFRu-GDC cathode with porous and thin Au paste.

Au paste is thin and porous, which ensures the transmission of electrons without hindering the transport of gases (Figure S5e).

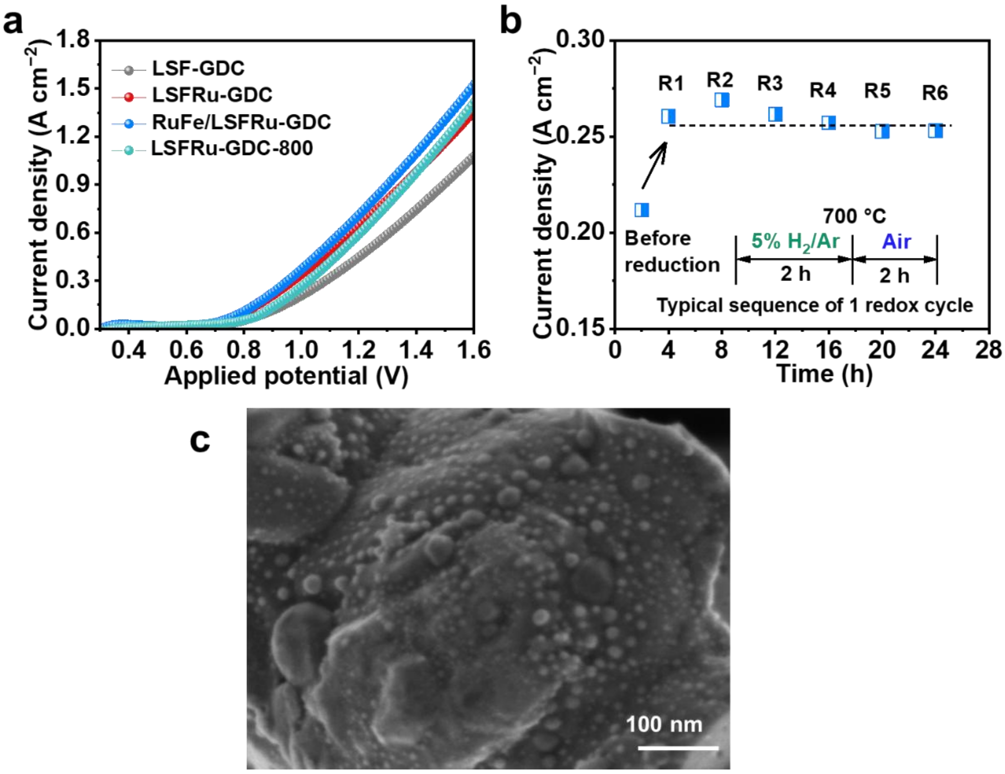

Figure S6. (a) Current density-voltage curves of LSF-GDC, LSFRu-GDC, RuFe/LSFRu-GDC, and LSFRu-GDC-800 based SOECs for CO_2 electrolysis at 800°C. (b) The redox cycling CO_2 electrolysis performance of LSFRu-GDC cell at 700°C. R represents reduction. (c) SEM image of LSFRu-800.

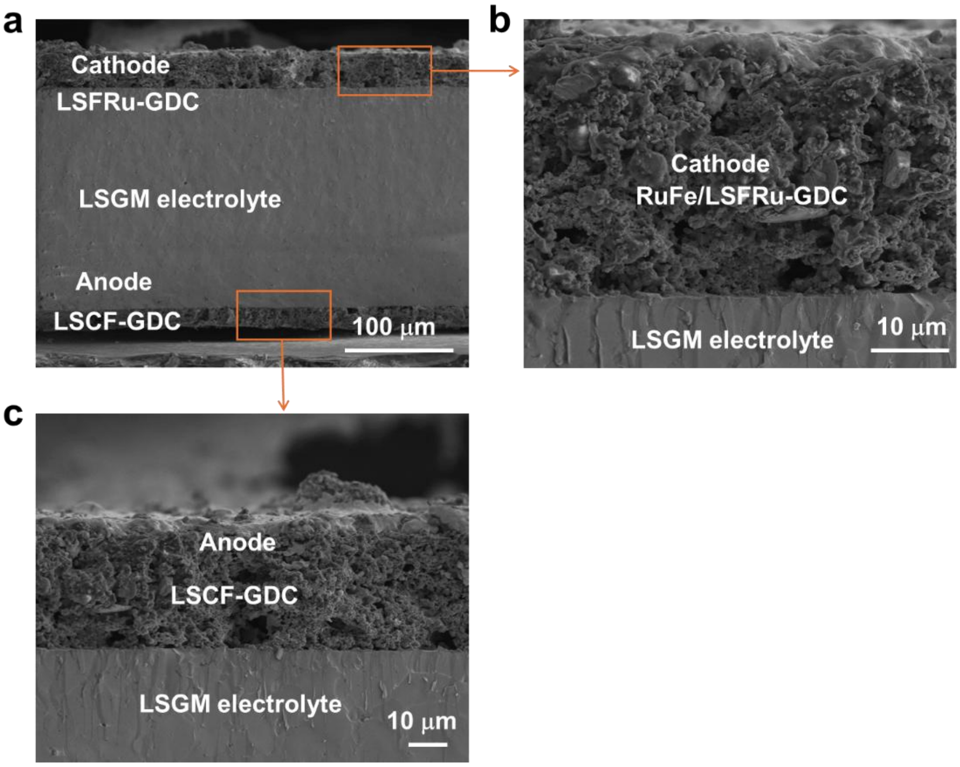

Figure S7. Cross-sectional SEM images of (a) LSFRu-GDC|LSGM|LSCF-GDC cell, (b) LSGM supported LSFRu-GDC cathode and (c) LSGM supported LSCF-GDC anode. Electrolyte thickness: ~200 μm.

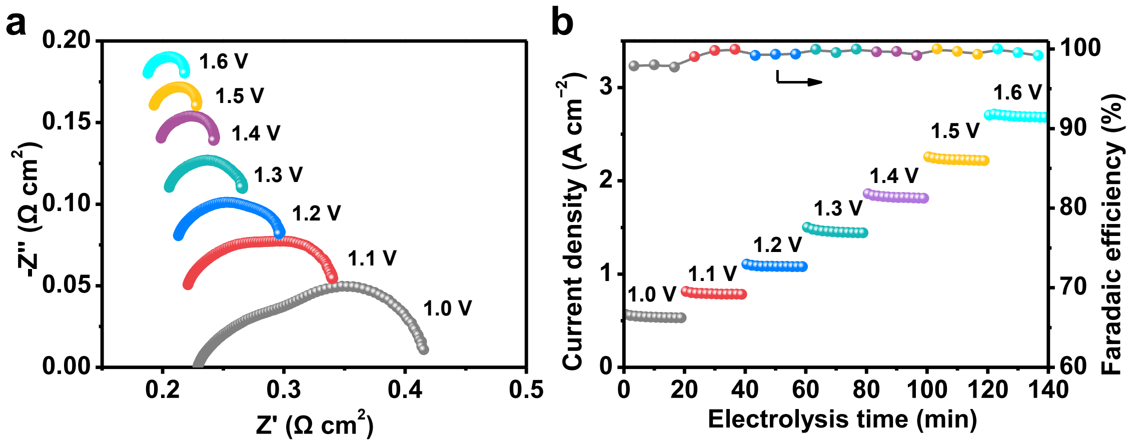

Figure S8. (a-b) Nyquist plots and short-term stability of CO_2 electrolysis of RuFe/LSFRu based cell under various applied voltages at 800°C, LSGM electrolyte: ~200 μm, CO_2: 50 mL cm^{-2}.

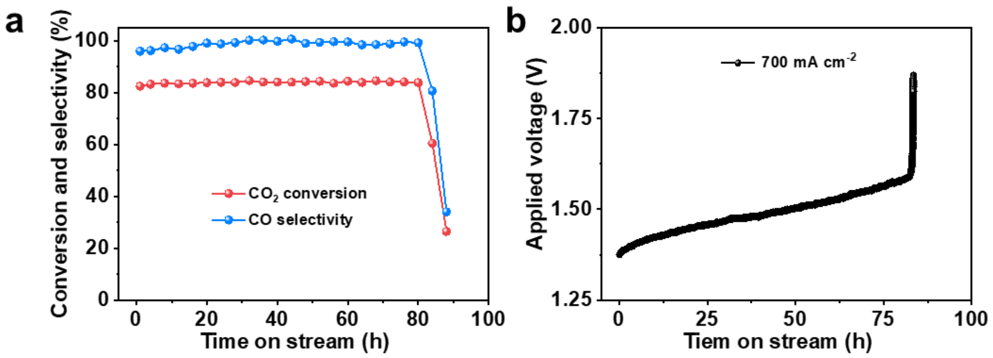

Figure S9. (a) Time-dependent CO_2 conversion and CO Faradaic efficiency at a constant current density of 700 mA cm^{-2}. (b) Time-dependent applied voltage of RuFe/LSFRu based SOEC for CO_2 electrolysis under 700 mA cm^{-2} at 800°C.

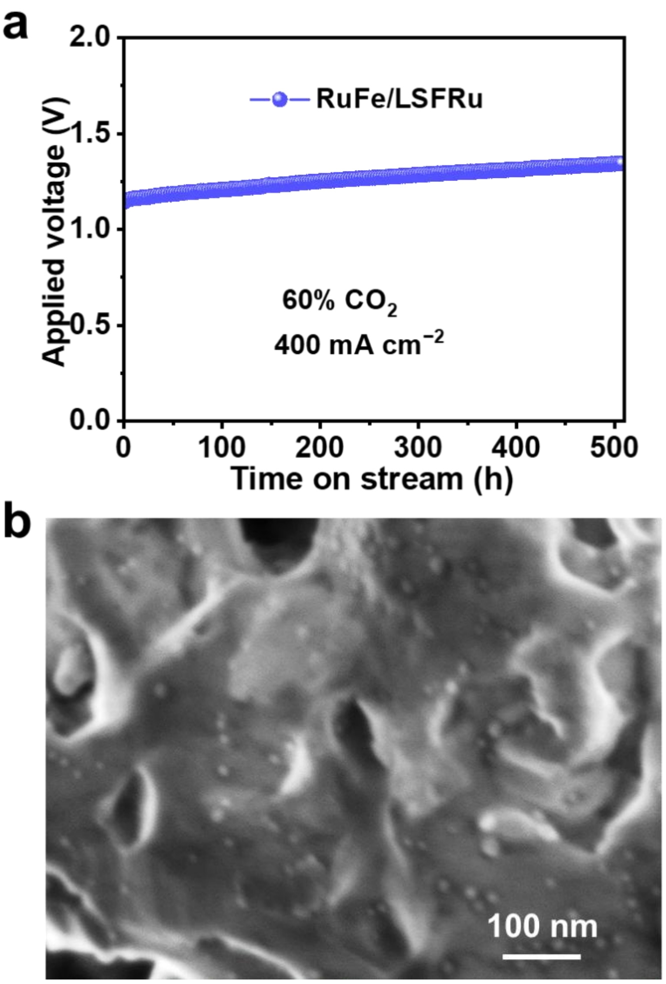

Figure S10. (a) Time-dependent applied voltage of RuFe/LSFRu based SOEC for CO_2 electrolysis under 400 mA cm^{-2} at 800°C. (b) SEM image of RuFe/LSFRu sample after 520 h stability test for direct CO_2 electrolysis.

Figure S10a shows the variation of applied voltage when directly electrolyzing CO_2 using SOEC in constant current mode (800°C, 400 mA cm^{-2}, 60% CO_2 in Ar, 5 mL min^{-1}), and the corresponding CO production is shown in Figure 3f. During CO_2 electrolysis at constant current, the applied voltage shows a slow upward trend, which indicates that the cell is slowly deactivating. The reasons for deactivation include e.g. element segregation in the oxygen electrode, element diffusion between the electrode and electrolyte, and reduced cathode catalyst sites.

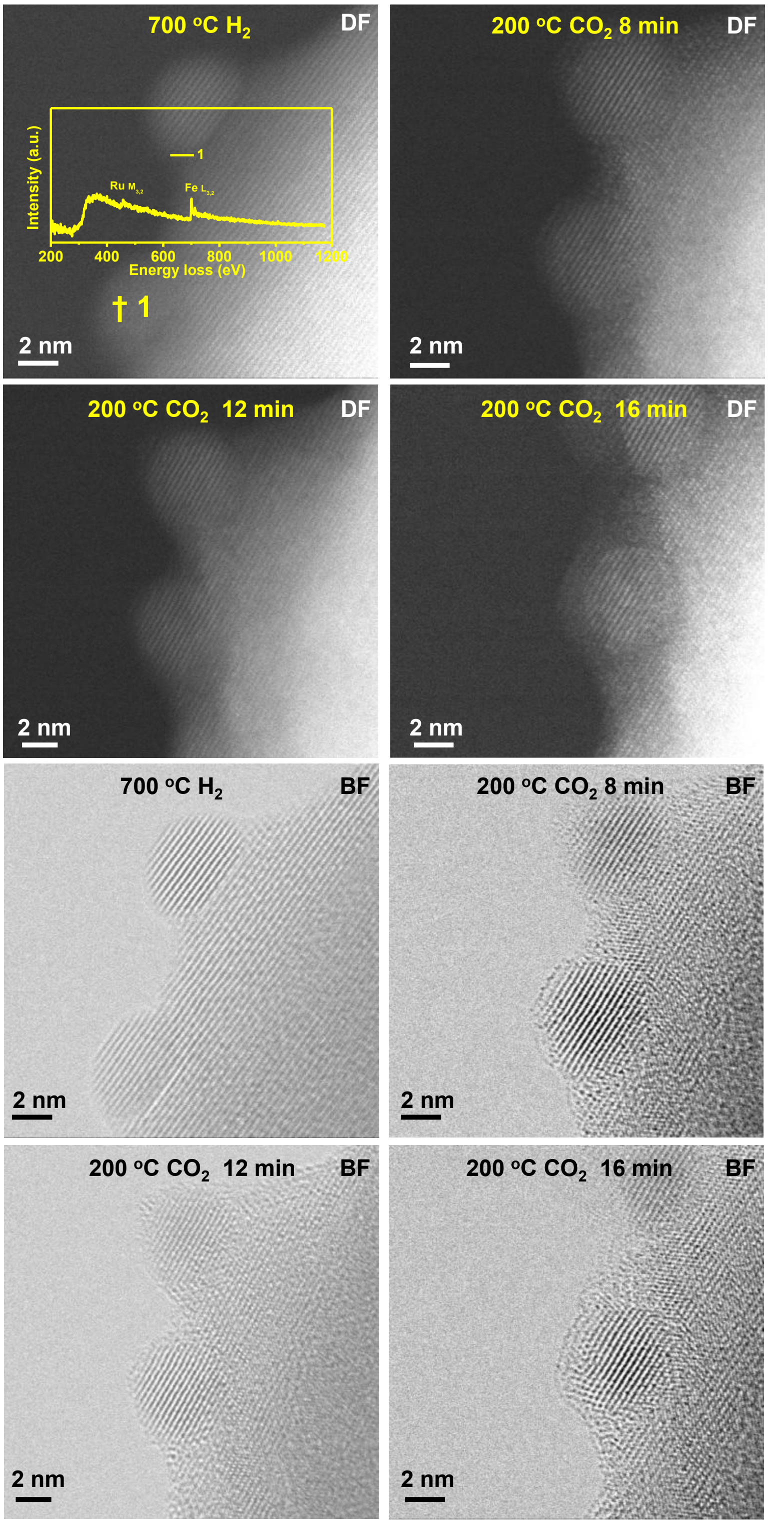

Figure S11. (a) *In situ* DF-STEM images of the exsolved RuFe/LSFRu interfaces during exposure of CO_2 at 200 °C. (b) *In situ* BF-STEM images of the exsolved RuFe/LSFRu interfaces during exposure of CO_2 at 200 °C.

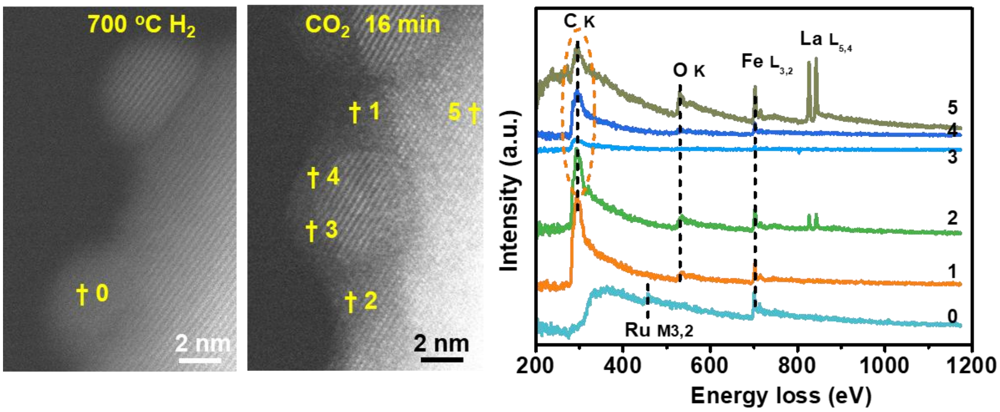

Figure S12. *In situ* BF-STEM images and STEM-EELS spectra of the RuFe/LSFRu interface after CO_2 treatment in Figure 3g.

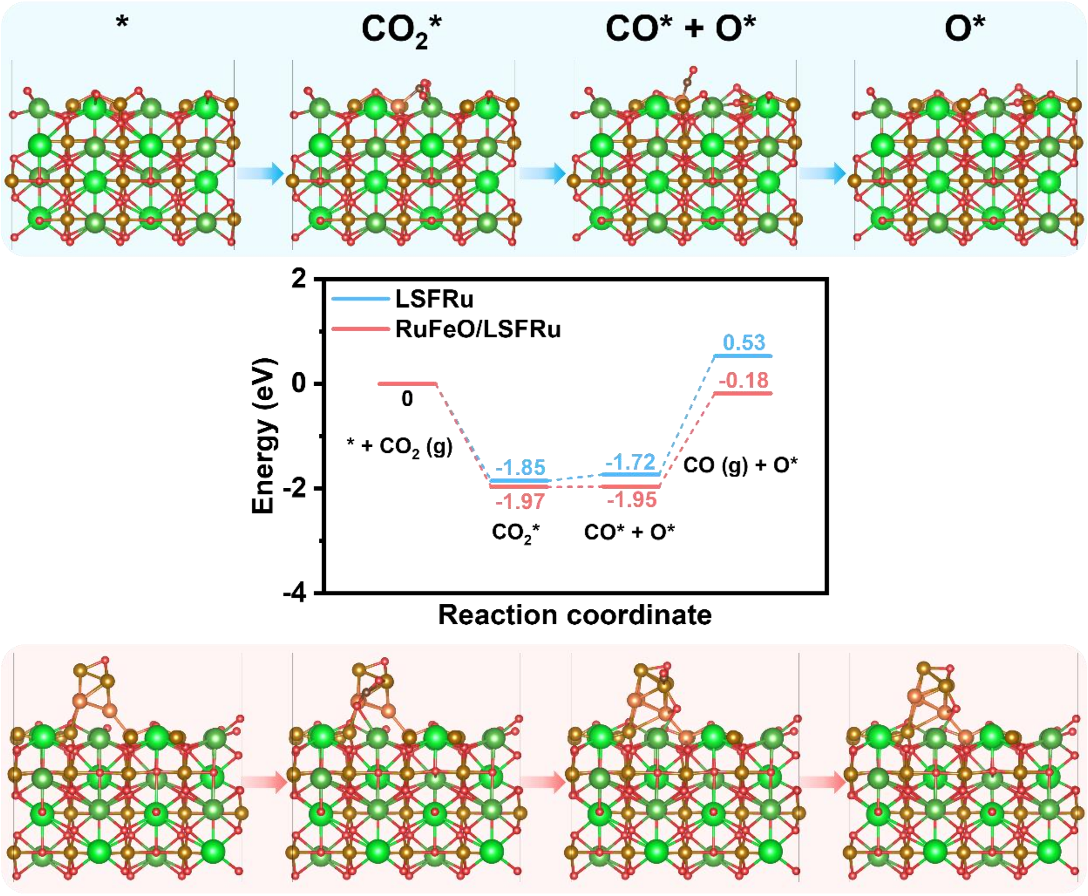

Figure S13. The energy profiles and the side views of optimized geometric structures of CO_2 activation at the oxygen vacancy of LSFRu (top) and the interface of RuFeO/LSFRu (down). La, Sr, Fe, Ru, C and O atoms are labelled by dark green, green, yellow, orange, brown and red balls, respectively.

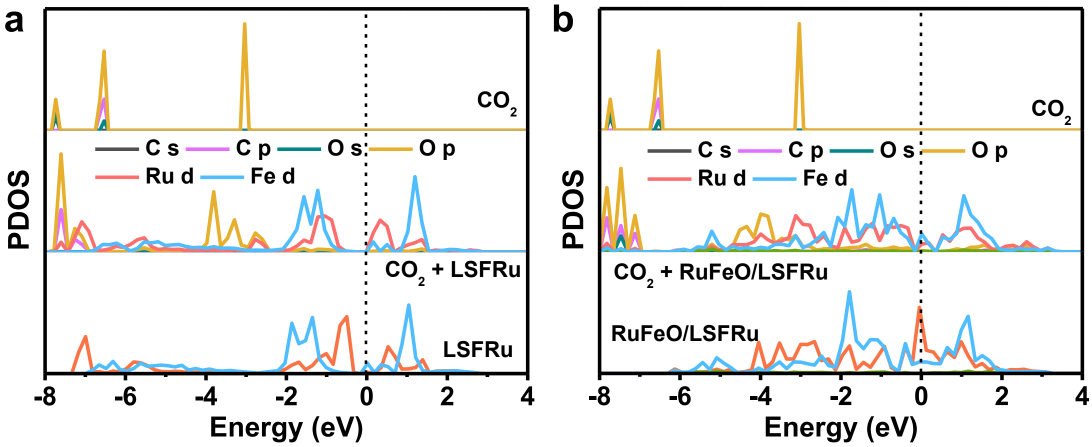

Figure S14. The projected density of states of CO_2, supports and their interactions with CO_2 adsorption at (a) the LSFRu perovskite surface with oxygen vacancy and (b) the interface of RuFeO/LSFRu. The Fermi level of the support (LSFRu and RuFeO/LSFRu) is set to 0 eV.

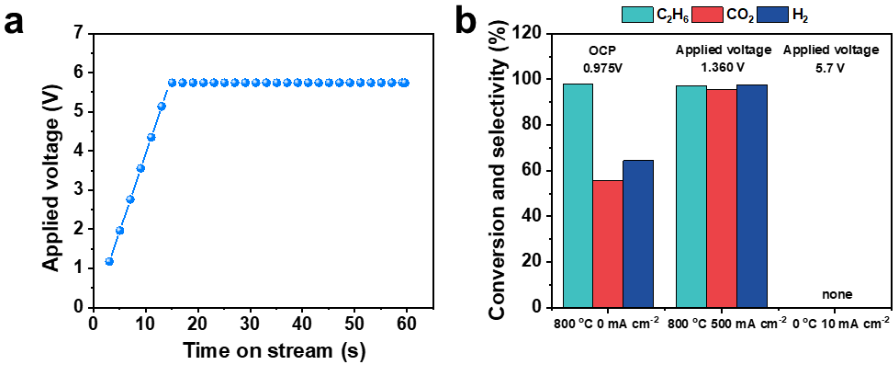

Figure S15. (a) The applied voltage of the purely electro-catalysis test (room temperature). (b) Performance comparison between purely thermal, purely electrochemical, and electro-thermal coupled modes.

To provide a direct performance comparison between purely thermal, purely electrochemical, and coupled modes, we conducted additional purely electro-catalysis tests. Due to the reaction temperature being at room temperature, no ethane reforming process occurred, meaning that no CO and H_2 products were detected. Meanwhile, oxygen ions can only migrate through the electrolyte at high temperatures in a solid oxide electrolysis cell. Therefore, the voltage reached the limit of the electrochemical workstation when a current density of 10 mA cm^{-2} was applied to the cell (Figure S15a). Similarly, no products were detected due to the lack of oxygen ion conductivity at room temperature (Figure S15b).

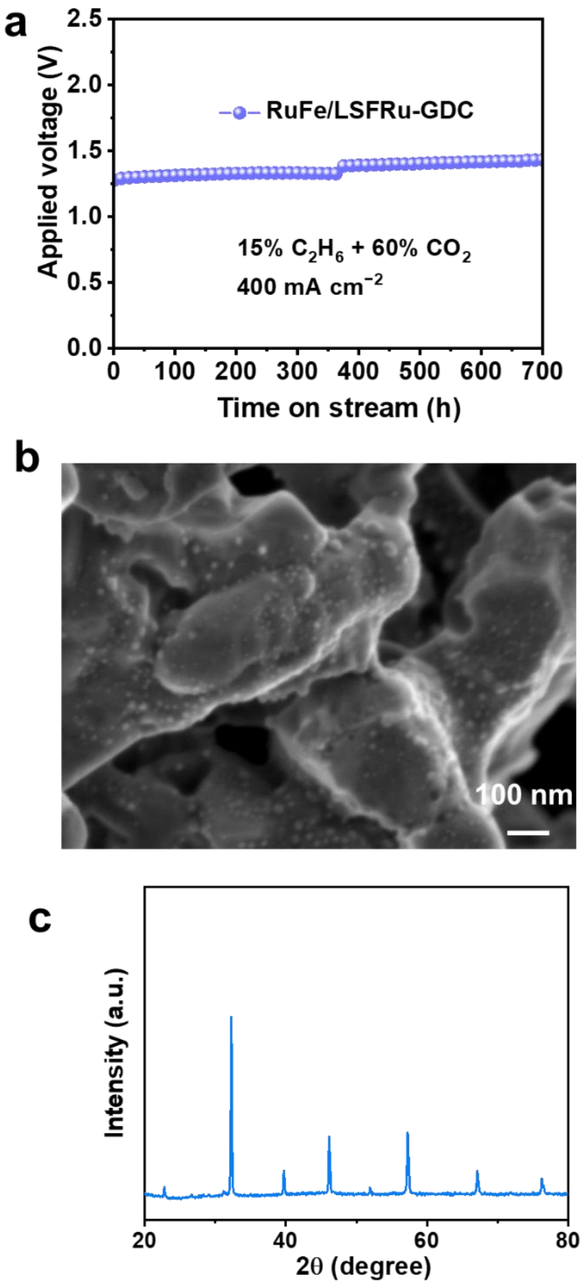

Figure S16. (a) Long-term tandem electro-thermocatalytic DER stability test of RuFe/LSFRu based cell under 400 mA cm^{-2} at 800°C. (b-c) SEM image and XRD patterns of RuFe/LSFRu sample after 700 h stability test for tandem electro-thermocatalytic DER reaction.

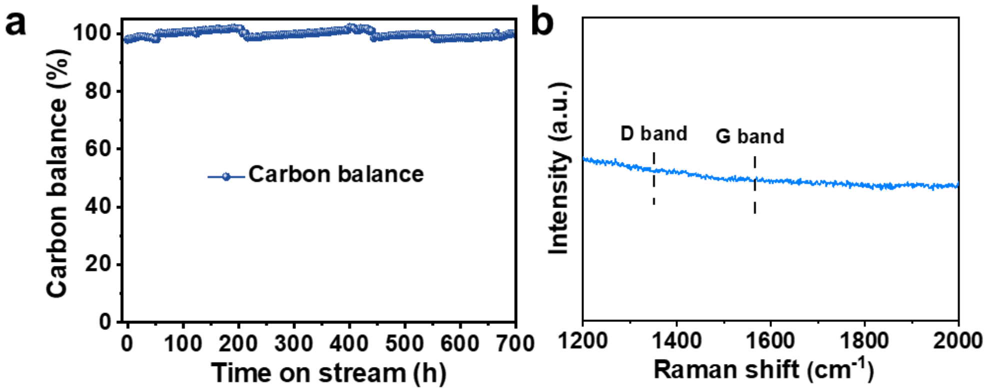

Figure S17. (a) Carbon balance of RuFe/LSFRu based cell during 700 h reaction at 800°C. (b) Raman spectrum of RuFe/LSFRu cathode after 700 h reaction at 800°C.

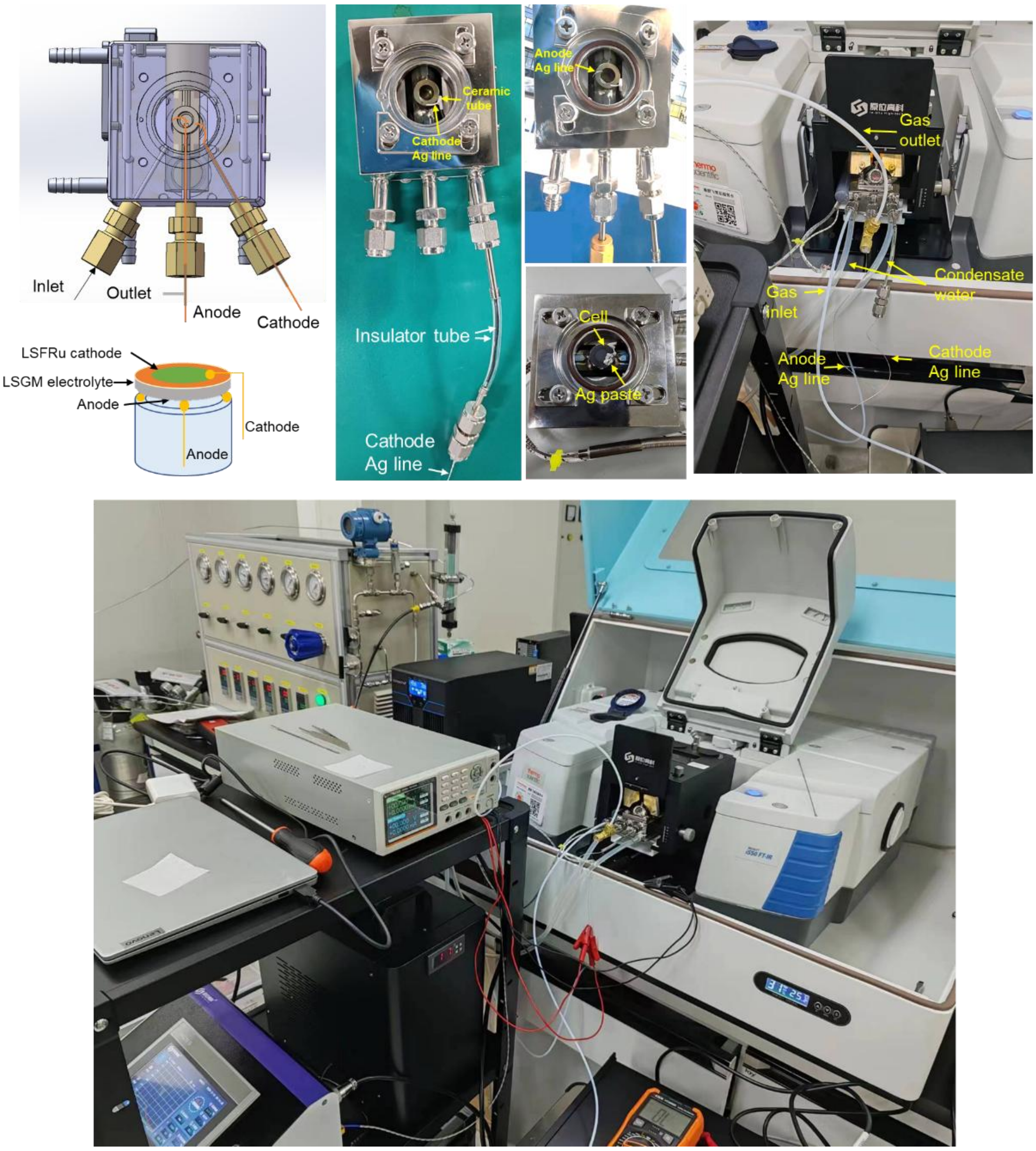

Figure S18. Schematic diagram and photograph of the *in situ* electrochemical DRIFTS experiments.

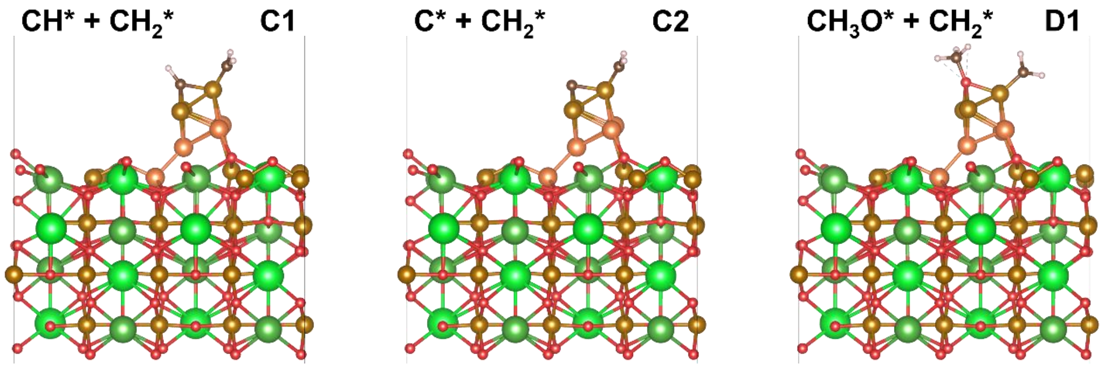

Figure S19. The side views of the optimized geometric structures of C_2H_6 activation to produce CO on RuFe-site of RuFe/LSFRu. La, Sr, Fe, Ru, C, H and O atoms are labelled by dark green, green, yellow, orange, brown, white and red balls, respectively.

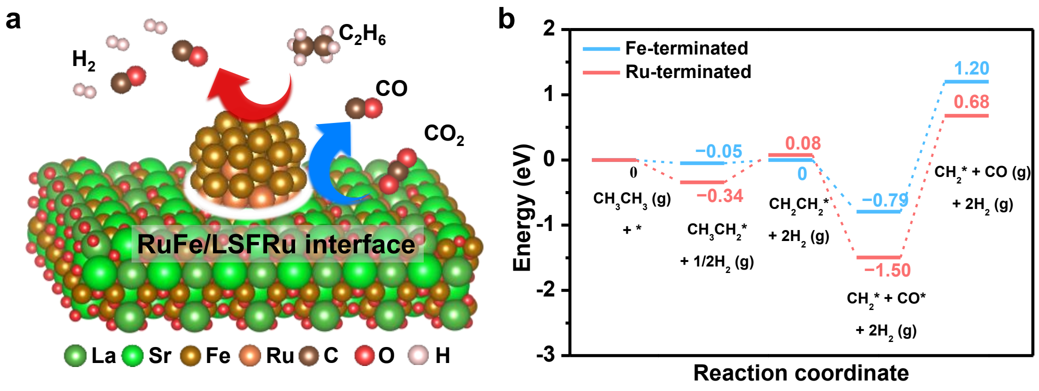

Figure S20. (a) Schematic illustration for the DER process at the RuFe/LSFRu catalyst. La, Sr, Fe, Ru, C and O atoms are labelled by dark green, green, yellow, orange, brown and red balls, respectively. (b) The energy profiles of C_2H_6 activation to produce CO on RuFe-site of RuFe/LSFRu catalyst with the Fe-terminated surface and Ru-terminated surface.

The RuFe/LSFRu catalyst with Fe atoms segregated to the surface is 0.41 eV lower in total energy than the Ru-terminated counterpart, indicative of Fe surface enrichment during CO_2 activation and the tandem electro-thermocatalytic DER reaction. Moreover, the Fe-terminated RuFe/LSFRu surface exhibits lower C-H bond dissociation barrier (0.05 eV *vs.* 0.42 eV) and CO desorption energy (1.99 eV *vs.* 2.18 eV) than the Ru-terminated surface, which promotes the DER process.

Table S1 Comparison of CO_2 electrolysis performance of different catalysts.

Cathode	Applied Voltage (V)	Current density (A cm^{-2})	CO_2 conversion (%)	Electrolyte thickness (μm)	Reference
Fe-SFMN	1.6	1.13	4.3	250	Nat. Commun., 2022, 13, 4618.
CoFe/SFMC	1.6	1.20	9.0	240	Adv. Mater., 2020, 32, 1906193.
SFTCMM	1.5	1.50	11.3	230	ACS Appl. Mater. Interfaces 2023, 15, 45905.
SFVMNT	1.5	1.66	20.7	210	J. Mater. Chem. A, 2024, 12, 18182.
FeCu/SFMCu	1.5	1.73	13.0	300	Sep. Purif. Technol., 2025, 363, 132143.
PCFN95	1.5	1.76	22.0	300	Energy Environ. Mater., 2024, 7, e12715.
FeRu/STFR	1.6	1.77	22.1	200	J. Power Sources 2024, 615, 235087.
LSFCNMM-F	1.5	1.78	33.3	230	J. Power Sources 2025, 634, 236498.
PLSBSCF	1.6	2.2	20.6	230	ACS Energy Lett., 2024, 9, 3818.
FeRu/SFRuM	1.6	2.25	16.9	260	Nat. Commun., 2021, 12, 5665.
CoFe/LSCFM	1.6	2.40	18	260	Angew. Chem. Int. Ed., 2020, 59, 15968.
RuFe/LSFRu	1.6	**2.75**	21.7	200	**This work**
	1.4	0.75	**83.4**	600	
R-SFCuM	1.6	2.85	17.8	250	Adv. Energy Mater., 2022, 12, 2202175.
SFMN	1.6	2.91	21.4	150	J. Am. Chem. Soc., 2025, 147, 21003

Fe-SFMN: Fe-$Sr_2Fe_{1.3}Ni_{0.2}Mo_{0.5}O_6$; CoFe/SFMC: CoFe/$Sr_2Fe_{1.3}Co_{0.2}Mo_{0.5}O_{6-\delta}$; SFTCMM: $Sr_2Fe_{1.0}Ti_{0.25}Cr_{0.25}Mn_{0.25}Mo_{0.25}O_{6-\delta}$; SFVMNT: $Sr_2Fe_{0.4}V_{0.4}Mo_{0.4}Ni_{0.4}Ti_{0.6}O_{6-\delta}$; FeCu/SFMCu: $Sr_{1.9}Fe_{1.5}Mo_{0.3}Cu_{0.2}O_{6-\delta}$; PCFN95: $(Pr_{0.4}Ca_{0.6})_{0.95}Fe_{0.8}Ni_{0.2}O_{3-\delta}$; FeRu/STFR: $Sr(Ti_{0\cdot3}Fe_{0\cdot63}Ru_{0.07})O_{3-\delta}$; LSFCNMM-F: $La_{0.6}Sr_{0.4}Fe_{0.3}Co_{0.2}Ni_{0.2}Mn_{0.2}Mo_{0.1}O_{3-\delta}$-F; PLSBSCF: $Pr_{1/6}La_{1/6}Sm_{1/6}Ba_{1/6}Sr_{1/6}Ca_{1/6}FeO_{3-\delta}$; FeRu/SFRuM: FeRu/$Sr_2Fe_{1.4}Ru_{0.1}Mo_{0.5}O_{6-\delta}$; CoFe/LSCFM: CoFe/$La_{0.4}Sr_{0.6}Co_{0.3}Fe_{0.7}Mo_{0.1}O_{3-\delta}$; R-SFCuM: $Sr_2Fe_{1.5}Mo_{0.3}Cu_{0.2}O_{6-\delta}$; SFMN: $Sr_2Fe_{1.3}Ni_{0.2}Mo_{0.5}O_6$.

References:

[1] G. Kresse, J. Furthmuller, *Computational Materials Science* **1996**, *6*, 15-50.
[2] P. E. Blöchl, *Phys. Rev. B* **1994**, *50*, 17953-17979.
[3] J. P. Perdew, K. Burke, M. Ernzerhof, *Phys. Rev. Lett.* **1996**, *77*, 3865-3868.
[4] G. Kresse, D. Joubert, *Phys. Rev. B* **1999**, *59*, 1758-1775.
[5] H. Monkhorst, J. Pack, *Phys. Rev. B* **1976**, *13*, 5188-5192.
